# Supplementary material for: Information sharing challenges in end-of-life care: a qualitative study of patient, family and professional perspectives on the potential of an Electronic Palliative Care Co-ordination System
Source: BMJ Open. 2020 Oct 5;10(10):e037483. doi: 10.1136/bmjopen-2020-037483 (PMC7537426; doi:10.1136/bmjopen-2020-037483)
Supplement: Supplementary data [file bmjopen-2020-037483supp001.pdf]

May 2018

Interview Topic Guide- Health and social care professionals

Please note the interview topic guide is a living document. The design is iterative to tailor the interview to the needs of the participant, and the issues that they feel are most important. As analysis occurs concurrently the topic guide is likely to be adapted over time - questions might be added, amended or omitted – and used flexibly.

Section A: for interview

1. **Introduction**

- Thank you for agreeing to take part in this focus group. As you know, we are talking to health and social care professionals about their understandings, views and experiences of end of life care, and how it is managed.
- With your permission I would like to record the interview; all details will be confidential.
- Do you have any questions or concerns?
- *Obtain written consent.*

2. Management of end of life care

Could you talk me through how end of life care is typically managed?

Which health (and social) care professionals are involved?

Do all GPs have contact with palliative patients? How often? What is the role/responsibility of palliative leads?

(Gain insight into IT system: EMIS or SystmOne. Why this one?)

Our understanding is that 'palliative care' is when a terminal diagnosis has been given, and 'end of life care' refers to the last 3 days of life. Does this fit with your understanding or would you use other terminology?

What does end of life mean to you? Is there a difference between "approaching" end of life and 'the end'? How uncertain is this?

How is end of life care shared, communicated and co-ordinated between different health and social care professionals?

How does information flow between these groups?

At any point does this information flow feel particularly challenging?

What systems do you use in the delivery of end of life care?

How much can other professionals see/interact with the data you record?

How much can you see/interact with data recorded by others?

From your perspective, what works well/not so well in the pathway?

How does this vary depending on where the patient is? (Home/hospice/care home/hospital)

How successful do you feel the current system is in facilitating the patient's end of life wishes/preferences?

Example of a recent case where you feel it was handled well/not so well

How confident are you in the management of end of life care?

How confident are you in engaging patients, and their families, in discussions around end of life care?

Standing H, et al. BMJ Open 2020; 10:e037483. doi: 10.1136/bmjopen-2020-037483

Is there anything that would improve your confidence in discussing and managing end of life issues?

3. **Sharing of information**

Are you familiar with the deciding right documentation?
How frequently do you come into contact with these documents?

How familiar are you with emergency healthcare plans?
Who completes these?
What kind of information is recorded?

What patient health data would be useful for you when managing a patient at the end of life?
(e.g. recorded by others at other sites)

Are there any issues that you currently encounter related to patient health data (regarding access/consent etc.)?
What are the barriers/limitations in terms of systems/patient perceptions?

4. **Electronic palliative care plan**

Have you heard of EPaCCs? What is your understanding of this plan?

*****SHOW EPaCCS VIDEO*****

How do you think this care plan might integrate into current service provision?
Can you foresee any barriers? (e.g. usability/resources/time/confidentiality)
Explore access to/willingness to use mobile devices

What potential barriers/facilitators do you foresee related to the consenting and sharing of patient health data specifically?

5. **Feedback**

- *Thank you for your time.*
- *What made you take part in this interview?*
- *Are we asking the right questions?*
- *Do these questions allow you to talk about the most important issues for you?*
- *Is there anything else you think it would be useful for us to know?*

Standing H, *et al. BMJ Open* 2020; 10:e037483. doi: 10.1136/bmjopen-2020-037483
