# Supplementary material for: Information sharing challenges in end-of-life care: a qualitative study of patient, family and professional perspectives on the potential of an Electronic Palliative Care Co-ordination System
Source: BMJ Open. 2020 Oct 5;10(10):e037483. doi: 10.1136/bmjopen-2020-037483 (PMC7537426; doi:10.1136/bmjopen-2020-037483)
Supplement: Author's manuscript [file bmjopen-2020-037483.draft_revisions.pdf]

BMJ Open is committed to open peer review. As part of this commitment we make the peer review history of every article we publish publicly available.

When an article is published we post the peer reviewers' comments and the authors' responses online. We also post the versions of the paper that were used during peer review. These are the versions that the peer review comments apply to.

The versions of the paper that follow are the versions that were submitted during the peer review process. They are not the versions of record or the final published versions. They should not be cited or distributed as the published version of this manuscript.

BMJ Open is an open access journal and the full, final, typeset and author-corrected version of record of the manuscript is available on our site with no access controls, subscription charges or pay-per-view fees (http://bmjopen.bmj.com).

If you have any questions on BMJ Open's open peer review process please email info.bmjopen@bmj.com

BMJ Open

Information sharing challenges in end-of-life care and the potential for an EPACCS: patient, family and professional perspectives

Journal:	*BMJ Open*
Manuscript ID	bmjopen-2020-037483
Article Type:	Original research
Date Submitted by the Author:	04-Feb-2020

Complete List of Authors:	Standing, Holly ; Northumbria University, Faculty of Health and Life Sciences<br>Patterson, Rebecca; Northumbria University, Research and Innovation Services<br>Lee, Mark; St Benedict's Hopsice and Specialist Palliative Care Centre<br>Dalkin, Sonia; Northumbria University, Faculty of Health and Life Sciences<br>Lhussier, Monique; Northumbria University, School of Health, Education and Community Studies<br>Bate, Angela; Northumbria University, Nursing, Midwifery and Health<br>Exley, Catherine; Newcastle University,<br>Brittain, Katie; Northumbria University, Department of Nursing, Midwifery & Health

Keywords:	Organisation of health services < HEALTH SERVICES ADMINISTRATION & MANAGEMENT, PALLIATIVE CARE, QUALITATIVE RESEARCH, Information technology < BIOTECHNOLOGY & BIOINFORMATICS

SCHOLARONE™
Manuscripts

I, the Submitting Author has the right to grant and does grant on behalf of all authors of the Work (as defined in the below author licence), an exclusive licence and/or a non-exclusive licence for contributions from authors who are: i) UK Crown employees; ii) where BMJ has agreed a CC-BY licence shall apply, and/or iii) in accordance with the terms applicable for US Federal Government officers or employees acting as part of their official duties; on a worldwide, perpetual, irrevocable, royalty-free basis to BMJ Publishing Group Ltd ("BMJ") its licensees and where the relevant Journal is co-owned by BMJ to the co-owners of the Journal, to publish the Work in this journal and any other BMJ products and to exploit all rights, as set out in our licence.

The Submitting Author accepts and understands that any supply made under these terms is made by BMJ to the Submitting Author unless you are acting as an employee on behalf of your employer or a postgraduate student of an affiliated institution which is paying any applicable article publishing charge ("APC") for Open Access articles. Where the Submitting Author wishes to make the Work available on an Open Access basis (and intends to pay the relevant APC), the terms of reuse of such Open Access shall be governed by a Creative Commons licence – details of these licences and which Creative Commons licence will apply to this Work are set out in our licence referred to above.

Other than as permitted in any relevant BMJ Author's Self Archiving Policies, I confirm this Work has not been accepted for publication elsewhere, is not being considered for publication elsewhere and does not duplicate material already published. I confirm all authors consent to publication of this Work and authorise the granting of this licence.

For peer review only

Information sharing challenges in end-of-life care and the potential for an EPaCCS: patient, family and professional perspectives

Holly C Standing[1], Rebecca Patterson[2], Mark Lee[3], Sonia Michelle Dalkin[4], Monique Lhussier[4], Angela Bate[1], Catherine Exley[5], and Katie Brittain[1]

[1]Department of Nursing, Midwifery and Health, Faculty of Health and Life Sciences, Northumbria University

[2]Research and Innovation Services, Northumbria University

[3]St Benedict's Hospice and Specialist Palliative Care Centre, Sunderland

[4]Department of Social Work, Education and Community Wellbeing, Faculty of Health and Life Sciences, Northumbria University

[5]Faculty of Medical Sciences, Newcastle University

Corresponding author: Dr Holly C Standing, Northumbria University, Coach Lane Campus West, Newcastle Upon Tyne, NE77AX (holly.standing@northumbria.ac.uk) 0191 232 6002

Word count 4,166

Abstract

Objectives To explore current challenges in inter-disciplinary management of end-of-life care in the community, and the potential of an Electronic Palliative Care Coordination System (EPaCCS) to facilitate the delivery of care which meets patient preferences.

Design Qualitative study using interviews and focus-groups.

Setting Health and Social Care Services in the North of England.

Participants 71 participants, 62 health and social care professionals, 9 patients and family members.

Results Four key themes were identified: information sharing challenges; information sharing systems; perceived benefits of an EPaCCS; barriers to use and requirements for an

For peer review only

EPaCCS. Challenges in sharing information were a source of frustration for health and social care professionals as well as patients, and were suggested to result in inappropriate hospital admissions. Current systems were perceived by participants to not work well - paper advance care planning documentation was often unavailable or inaccessible, meaning it could not be used to inform decision-making at the point of care. Participants acknowledged the benefits of an EPaCCS to facilitate information sharing, however they also raised concerns about confidentiality, and availability of the increased time and resources required to access and maintain such a system.

Conclusions EPaCCS offer a potential solution to information sharing challenges in end-of-life care, however, successful long-term use of the system requires investment in training and resources. There is a need for further qualitative research evaluating use of an EPaCCS, which explores benefits and challenges, uptake and reasons for disparities in use to better understand the potential utility and implications of such systems.

Keywords

Information technology, organisation of health services, palliative care, qualitative research

For peer review only

ARTICLE SUMMARY

Strengths and limitations of this study

- Large participant group from various stakeholder groups including health and social care practitioners, patients and carers
- This is the first study to include the experience of social workers and of a coroner

- Semi-structured interviews allowed us to collect rich descriptions of participants' experiences and perceptions of information sharing in end-of-life care and of an EPaCCS
- The study was conducted by a team of experienced researchers from a range of disciplinary backgrounds

INTRODUCTION

The development of end-of-life care guidance in recent years supports attempts to allow dying persons more control over their end-of-life experience, through engagement of patients in Advance Care Planning (ACP).[1] ACP has been defined as 'a process that supports adults at any age or stage of health in understanding and sharing their personal values, life goals, and preferences regarding future medical care'. The goal of advance care planning is to help ensure that people receive medical care that is consistent with their values, goals and preferences during serious and chronic illness.'[2] ACP can include anyone involved in the provision of the patient's care, including nurses, doctors, care home managers and family members. Documents completed as part of ACP can include: a do not attempt cardiopulmonary resuscitation order (DNACPR), emergency health care plan (EHCP), and a written summary of the patients care preferences.[3] Paper versions of this documentation are completed and held by the patient to present to the health and social care professionals they encounter.[4] ACP can facilitate the delivery of care which better reflects the patient's personal preferences,[1] and can have positive impacts on the person at the end-of-life and their family.[5] However, evidence suggests that the completion and use of ACP documentation is inconsistent.[6 7]

The delivery of high quality end-of-life care is an inter-professional endeavour, where the resources of various health and social care professionals must be combined for the benefit of

For peer review only

the patient.[8] To achieve this, efficient inter-professional communication and information sharing across health and social care professionals involved in community end-of-life care is essential[9], but presents many challenges.[10-12] Accessibility of patient information can be limited, particularly for services who may only be episodically involved in end-of-life care but that offer crucial and timely support, such as paramedics, out-of-hours doctors, Admiral nurses, care home staff, and social workers.[13 14] Poor (or lack of) access to patient information can effect professionals' confidence in managing end-of-life patients, [13 14] family members satisfaction with care, [15] and patient outcomes.[5] Sharing of patient information is made more challenging by inconsistent documentation practices and systems which vary across organisations and professional boundaries.[16]

Electronic record systems offer a potential solution to these information sharing challenges.[17] A Framework for Action published by the National Information Board in 2014, details 2020 as a deadline for digital, real-time, and interoperable health records to be rolled out within the NHS.[18] Electronic Palliative care Co-ordinating Systems (EPaCCS) are an example of such an innovation. EPaCCS is an umbrella term covering a number of different electronic solutions that aim to capture patient wishes and preferred place of death and improve co-ordination of care in real time, through enabling the sharing of information across health and social care services. A Department of Health pilot evaluation in England estimated that EPaCCS could save £133,200 in hospital admissions per annum per 200,000 patient population.[19] However, research indicates that professional uptake and patient registration on such electronic systems is currently very low.[20] Challenges surrounding the use of an EPaCCS include professional reluctance to initiate end-of-life discussions,[6 21] increased burden from data entry for the primary users[20], data ownership, funding, and consent.[22]

This article reports data from a project exploring the potential of an EPaCCS solution for improving information sharing and coordination in end-of-life care. We provide an in-depth

For peer review only

study of the professional and organisational factors that promote or inhibit the acceptability, usefulness and integration of collaborative care planning across health and social care into service delivery and everyday practice.[23-25]

METHODS

This study employed qualitative methods, involving semi-structured interviews and focus groups with patients, bereaved family members, and health and social care professionals.

Participants

A heterogeneous purposive sampling approach was employed to ensure representation from all health and social care professional groups who have a potential role in end-of-life care, and that a variety of perspectives and experiences of end-of-life care were sampled. Ethical approval for this study was obtained from the Health Research Authority (REC reference: 17/LO/2100).

Data collection

An interview schedule was developed by HS, RP and KB which included: current approaches to end-of-life care management, access to patient data, and perspectives on an EPaCCS (including perceived benefits and challenges). The interview schedule evolved during the data collection process to allow iterative data analysis and further exploration of themes derived from the data. Informed consent was given by all participants prior to taking part. Interviews were digitally recorded.

Participants were recruited from the North of England. Data collection took place from March to October 2018, interviews and focus groups were conducted at the participants' place of work or home, at university or community venues, or over the telephone, according to participant preference. Interviews and focus groups were conducted by HS, RP and KB.

For peer review only

Table 1 details participant demographics. Clinician interviews ranged from 28-93 minutes, focus groups from 39-63 minutes, patients and family member interviews from 22 and 80 minutes. We endeavoured to engage in a process of reflexivity throughout the project. Field notes were collected before, during and after data collection. Data has been pseudonymised to protect participant identity.

Table 1: Participant demographics

**Health and social care professional role**	**No.**
GP	7
Out of hours GP	2
Nurse (including: Macmillan, Marie Curie, care home, district, rapid response, Admiral nurse)	21
Formal carer (care home)	3
Paramedic	10
Social worker	6
Pharmacist	4
Hospital doctor	4
Other supporting professions (including: care coordinator, Macmillan manager, physiotherapist, coroner, palliative care team)	5
**Patient/family**	
Patient	7 (4 female, 5 lived alone, 6 60+ years old)
Family	2 (both female, 60+)
**Total**	**71**

For peer review only

Data Analysis

Audio recordings were transcribed verbatim by an independent transcription company; transcripts were checked for accuracy by (HS, RP, KB). The data management software NVivo 12 was used to develop and refine a coding scheme.

We adopted an iterative approach to data analysis. Data collection and analysis ran concurrently throughout the study and analysis of early transcripts informed the refinement of the interview schedule for later interviews. The five-step process of thematic analysis [26] was adopted to develop and refine codes and themes from the data. Field notes taken during data collection were utilised in data analysis to enhance the reflexive process. Credibility measures included a process of continuous review and discussion of themes at group analysis sessions, and at wider team meetings. Three researchers (HS, RP, KB) independently coded 20% of the transcripts before coming together to compare codes and discuss discrepancies.

Patient and public involvement

The study was supported by a patient and public involvement representative who contributed to the design and development of the study, advised on recruitment processes, and participant information and consent materials.

FINDINGS

Four themes were identified through data analysis: information sharing challenges; information sharing systems; perceived benefits of an EPaCCS; barriers to use and requirements for an EPaCCS. These are presented in turn below, with illustrative quotations.

Information sharing challenges

Management of end-of-life patients in the community is an inter-disciplinary issue, involving a range of different health and social care services including (but not limited to): GPs, nurses, social workers, paramedics, and out-of-hours GPs. Effective management of end-of-life patients living in the community requires efficient information sharing between these services.

Interviewees were keen for end-of-life patients in the community to be managed at home and avoid inappropriate hospital admissions where possible, however, poor communication and

For peer review only - http://bmjopen.bmj.com/site/about/guidelines.xhtml

information sharing between services was a barrier to achieving this goal. Paramedics and out-of-hours GPs may be alerted by call handlers that the patient they are attending is at the end-of-life, but often were going in 'blind' as they had no prior access to the patient's condition or care preferences. Without access to up-to-date patient information, paramedics felt that in such situations they had no choice but to admit the patient:

> *The number of times I've said, "They are going to have to go in. I don't want to take them in, but I don't have a choice here, because I don't have the information." If you don't have the information, you can't make an informed decision, so to leave them at home would be the wrong thing to do, even though you know morally you'd like to.* (Paramedic6)

Inappropriate hospital admissions and paramedic calls outs were suggested to result in negative impacts on the patient and their family, and were also suggested to be a waste of scarce NHS resources.

Inappropriate hospital admission of end-of-life patients was also a source of frustration for GPs who suggested that these could be avoided if they were contacted for advice:

> *I suppose the admissions where they're palliative are the ones that I get annoyed about [...] they get admitted, and if somebody had asked you, you know, "What should we do with this lady?"* [but]*that's not always easy because we don't work every day. We are obviously not working in the evenings or the weekends*[...] (GP6)

Indeed, the continuity of care afforded by the GP role means they often have established relationships with end-of-life patients and would most likely have access to detailed information regarding their condition, history and care preferences, which should inform

For peer review only

decision making in any circumstance. However, GPs are not available 24/7 and there is a need for patient information to be stored in a manner that is easily accessible to all services who may require it.

Poor communication and information sharing could have negative impacts on the patient and their family. Where patient information is unavailable, and services were going in ‘blind’ the burden of information sharing falls on the patient or their family.

> *Whatever doctor I would see, they wouldn't know a lot about me, so I'd have to constantly start from the beginning* […family] *couldn't come into the meetings with me because they'd get so upset because they'd watch me explain everything from the start. I think that was worse than hearing any bad news* (Patient5)

Having to repeatedly reiterate their story could be distressing for the patient and their family, and could also damage the professional patient relationship; as the patient above describes this constant repeating of her story led her family to disengage from encounters with health and social care professionals.

Lack of information sharing mean it is not always clear who should be responsible for verifying death and issuing the death certificate. This could result in the death being designated as ‘unexpected’ and the coroners being inappropriately involved, causing unnecessary delays and distress to the family:

> *when they’ve gone home expecting that* [the patient will die] *and then all of a sudden somebody says, “We don’t know who can issue the certificate because the GP hasn’t seen them because it was the out of hours doctor* [who came]…” *and it takes hours to sort it out*. (Coroner1)

For peer review only

Information sharing systems

Information about patients at the end-of-life is primarily stored in two formats: 1) paper-based ACP documents which are held by the patient, and 2) electronic patient record systems.

Paper ACP documents facilitate information sharing, however, their usefulness is largely dependent on the patient, or their family, having the documents ready to hand or easily accessible. Many interviewees indicated this was often not the case:

> *you're meant to have a bright yellow envelope on your mantle or on your sideboard. So, if you go in as a doctor, or if you go in as an ambulance crew, you know it's there, and it's bright, illuminous yellow, but the number of folks, "Oh, I've got one of those," and it's under four piles of stuff, or it's in their bedside cabinet. It's like, "Well, no-one's going to respect that," because no-one's going to spend 10 minutes looking for it if you've arrested. They're going to start resus.* (Out-of-hours GP1)

If the documentation is not available, either misplaced or forgotten, then health and social care professionals have to proceed as if it did not exist.

Having a standardised location for the documentation in the patient's home (such as the mantelpiece, for example) may improve access to this information. However, keeping the documentation in a visible location may also be a potential source of distress for families. Whilst some patient interviewees were comfortable having theirs visible, others chose to keep them hidden as they acted as a constant reminder of the imminent death.

> *I had put the yellow envelope in my knicker drawer* […] *They said, "Put it up there."* [mantel piece] *So, I said, "No, I'm not putting it up there. I don't want to look at that all the time."* (Bereaved family2)

For peer review only

In addition to paper versions of the documentation, all health and social care services utilise electronic patient record systems to record and manage the information that they collect. These systems tend to work in isolation, and are generally not inter-operable, meaning there is no simple way to share information with others. Many patients assumed that their information was being shared, and they did not necessarily perceive services as discrete organisations, rather part of a united healthcare system. Although patients recognised that protecting patient confidentially was important, the benefits of sharing information were perceived to significantly outweigh these concerns.

> *What if you travel? What if you're visiting round the country? You don't know what's going to happen* […] *I went to London a couple of times when I was ill. What happens if something happens to me down there? How long would it take them to find anything out about me? In the meantime, what's happening to me?* (Patient5)

Perceived benefits of an EPaCCS

Interviewees identified several potential benefits of adopting an EPaCCS. NHS information systems were perceived by the majority of interviewees, both patient and professional, as being extremely outdated.

> *I certainly think there should be a national set-up. Everybody should be reading from the same sheet, yes.* (Patient6)

Patients welcomed a system that would facilitate better transfer of information between services so that they would no longer have to act as the conduit for passing information between services by repeating their story.

For peer review only

Professionals indicated that an EPaCCS may save time and facilitate the provision of care that was better aligned with patients' wishes. An EPaCCS could potentially allow multiple users to simultaneously access and update a patient's record, ensuring changes to the patient's condition and/or care plan are immediately accessible to all professionals involved in their care.

> *I think the thing is, when it's a paper copy there's only one actual current live copy that is up to date* […] *the electronic copy means multiple users could be using it at the same time.* (Nurse6)

An EPaCCS was suggested to be particularly beneficial to services without sustained relationships with end-of-life patients who were often 'information poor'. Improved access to patient information could potentially increase the confidence of these services in delivering end-of-life care.

> *You do get very limited information and you're going into a person to talk to them about what care they need with very limited information. And sometimes when they are end-of-life, the last thing they want to talk about is how they're going to die and why they're going to die* (Social Worker5)

Barriers to use, and requirements for an EPaCCS

Professional interviewees did not express universal enthusiasm for the introduction of an EPaCCS. Paramedics and out-of-hours GPs were generally positive about the potential of the system, as discussed above, however GPs were less enthusiastic; the system was not necessarily perceived to offer improvements over current approaches to information storing and recording. GPs expressed concern about implications of the system for their workload;

For peer review only

GPs are often the most 'information rich' meaning responsibility for inputting data into the system would fall on them. If the EPaCCS was not fully inter-operable with existing systems it would result in duplication of tasks and increased demands on GP time.

> If it's [a] *separate* [system] *then you'd be duplicating because you'd need to add it to the system wouldn't you?* […] *You'd find GPs wouldn't engage with it, because it would just be an extra thing they have to do.* (GP3)

Data protection and the security of patient information was a major concern for many of the professional interviewees.

> *We're still recovering from a cyber-attack a year ago, and there are still some documents that we can't open* […] *so it has to be encrypted to a level that it's safe* (Hospital doctor3)

Such concerns are understandable given recent security threats [27] and scandals involving misuse of personal data.[28] However, this did not appear to be a concern for patients, many of whom assumed that data sharing practices already occurred. Moves to facilitate information sharing through electronic records with the aim of improved care were seen as welcome and necessary progress.

Incorporating the informational requirements of all health and social care groups into one system was highlighted as a challenge; different roles have different informational needs. For example, nurses indicated that lists of patient medication are essential information for their practice to ensure they are not prescribing contraindicated medications. Catering for individualised requirements such as these risks the EPaCCS becoming overwhelming and challenging to navigate.

For peer review only

It might just slow people down. It will take longer actually to get through cases because you have to read through them [patient notes] (Out-of-hours GP1)

Having to sift through irrelevant information to find what is germane to their role may become burdensome. An EPaCCS with extensive information about the patient and limited in its ability to filter efficiently may not be preferable to paper ACP documentation being available upon arrival.

Professionals who spend most of their time working remotely, doing home visits, may gain the most benefit from an EPaCCS.

electronic information, that would solve the main problems that we have, which is lack of information at times. So yes, that would be great. (Paramedic5)

However, an EPaCCS needs to be accessible to these professionals. If an EPaCCS cannot be accessed and updated by those working in the community, the information contained within will quickly cease to be up-to-date. Successful uptake of an EPaCCS is also dependent upon the technology available to access the system. Electronic devices available to those working in the community were reported to often be of low specification, raising doubts about likelihood that they would be able to support accessibility.

It also has to be an 'on the go' portal, you were saying how busy you are and so it has to work on a tablet or on an iPad because a lot of this work is being inputted in the community. You can't rely on someone going back to their office, after they've done five house calls or five social work visits or

For peer review only

> *five whatever* [..] *It has to be a real-time and easy to access.* (Hospital Doctor2)

A need for ongoing support for the EPaCCS following the implementation period was highlighted. Interviewees expressed concern that investing time learning a new system would be wasted if ongoing infrastructure was not available to support its continued use.

> *I've done many projects before within health, and there's a massive emphasis placed at the beginning of every project and they put it on. Then, afterwards, staff move away, because it's not part of their project and we're suddenly left with it. The system is changing and evolving, but there's not always the infrastructure in place to support us to update it. I think that's a real concern.* (Nurse19)

For peer review only

Implementation of an EPaCCS was not necessarily perceived to solve the challenges in inter-disciplinary management of end-of-life care alone. Access to ACP documentation will only be useful if the documentation itself is completed to a satisfactory standard, if insufficient detail is provided it cannot be used to inform decision making. Paramedics and out-of-hours doctors indicated that ACP documentation that they encountered was of a variable standard, and these documents should be completed with these services in mind as the intended reader.

> *I'm making a decision within 30 seconds I've walked through the door, "Am I going to attempt to save a life or am I going to let them expire?" The only thing between me and setting off a treatment path is the end-of-life care plan, so it needs to make perfect sense.*(Paramedic4)

DISCUSSION

Principle statement of findings

This study provides insight into current challenges in information sharing in end-of-life care, which were recognised as a barrier to effective end-of-life care, and the potential of an EPaCCS system to overcome these challenges. Inconsistent information sharing within and between health and social care services was a source of frustration for patients, paper based ACP documentation does not appear to be an effective system for recording and sharing patients' care preferences; often these documents are unavailable when required and are variable in quality. Patients welcomed the idea of an EPaCCS that facilitated sharing of their information between health and social care services, they did not report the same concerns about data protection and security issues that concerned clinicians. Both groups of interviewees perceived the potential for improvements in delivery aligned with patient's wishes from adoption of an EPaCCS. Perceived barriers to the successful implementation of an EPaCCS were focused on increased demand on time and the lack of infrastructure in place to support the system. Care must be taken to ensure that information contained within the EPaCCS does not become overwhelming. This is particularly important for emergency services, such as paramedics, who work under extreme time pressures limiting their ability to access and digest vast amounts of information. Introduction of an EPaCCS was not seen to resolve current challenges in management of patients at the end-of-life without investment into improving the frequency with which ACP documentation is completed and the quality of the information recorded. Paramedics in particular suggested their informational needs must be a priority to ensure ACP documentation is completed to a standard where it can be used to inform decision making.

For peer review only

Strengths and weaknesses

A key strength of this study is the large sample which includes a wide range of health and social care professionals involved in end-of-life care, the majority of previous qualitative studies have employed small samples,[29 30] and ours is the first study to our knowledge to include the perspectives of supporting professions such as social workers and coroners. In addition, our study included a greater number of interviewees from out-of-hours services than previous qualitative studies.[20 30] As the data were collected during the developmental pre-implementation phase of an EPaCCS trial, participants were only able to reflect hypothetically on the impacts of such a system on their practice rather than drawing on direct experiences of working with the system. In line with previous EPaCCS studies, a second limitation of the study stems from the recruitment process for patients and family members, which was, operationalised through GP practices. Staff thus acted as gatekeepers and may have been selective in who they referred for participation.

Comparison with other work

There are only a limited number of qualitative studies exploring attitudes towards and use of EPaCCS.[20 29-31] Our findings support those of a recent systematic review which identified the burden of inputting data and IT systems as the main challenge to implementation of EPaCCS.[32] GP interviewees expressed a reluctance to dedicate time to inputting data into the EPaCCS, this has been observed in previous studies where GPs have reported delegating much of this work to administrative staff.[20]

A key finding of our study was that introduction of an EPaCCS alone does not provide a solution to some of the current difficulties regarding inter-disciplinary management of end-of-life patients in the community. An EPaCCS will only facilitate access to patient information, the utility of which is largely dependent upon its quality; improving access to

For peer review only

ACP documentation is not going to improve care if the information recorded within them is of poor quality or insufficient detail, or has not been completed. This demonstrates a need for investment in training for health and social care professionals in ACP discussions and documentation to instil confidence and improve quality. Inconsistencies in ACP documentation have also been observed within an Australian healthcare context.[33]

This article highlights concerns regarding the infrastructure required to support an EPaCCS including: demand on already stretched professionals, technology provision required to enable access, and continued support to ensure the system is updated. Ensuring appropriate infrastructure is in place has emerged as a challenge in the implementation of coordinated records for end-of-life patients in both UK and US contexts.[32 34] As this article focuses on the pre-implementation stage of an EPaCCS trial future research should explore the long-term uptake and impact of an EPaCCS to better understand the infrastructure required to sustain such a system. High quality qualitative research that explores the benefits and challenges experienced whilst using the system for all stakeholders including in-hours and out-of-hours services, and patients is required. High and low users of the system should be explored to identify reasons for disparities and potential ways of promoting equitable use of the system.

Conclusions

Our study suggests that an EPaCCS may offer a potential solution to information sharing difficulties in the inter-disciplinary management of end-of-life care. However, successful uptake of an EPaCCS requires investment in training and resources.

REFERENCES

1. NICE. Advance Care Planning: Emergency and acute medical care in over 16s: service delivery and organisation. 2018
2. Sudore RL, Lum HD, You JJ, et al. Defining Advance Care Planning for Adults: A Consensus Definition From a Multidisciplinary Delphi Panel. *J Pain Symptom*

Manage 2017;53(5):821-32.e1. doi: 10.1016/j.jpainsymman.2016.12.331 [published Online First: 2017/01/08]

3. Northern Cancer Alliance. Deciding right Regional forms https://www.northerncanceralliance.nhs.uk/deciding-right/deciding-right-regional-forms/2019 [cited 2019 6th November]. Available from: https://www.northerncanceralliance.nhs.uk/deciding-right/deciding-right-regional-forms/ accessed 6th November 2019.
4. Mullick A, Martin J, Sallnow L. An introduction to advance care planning in practice. *Bmj* 2013;347:f6064.
5. Detering KM, Hancock AD, Reade MC, et al. The impact of advance care planning on end of life care in elderly patients: randomised controlled trial. *BMJ* 2010;340 doi: 10.1136/bmj.c1345
6. Dalkin SM, Lhussier M, Philipson P, et al. Reducing inequalities in care for patients with non-malignant diseases: Insights from a realist evaluation of an integrated palliative care pathway. *Palliative Medicine* 2016;30(7):690-97. doi: 10.1177/0269216315626352
7. National Institute for Health and Care Excellence. Chapter 15 Advance Care Planning. Emergency and acute medical care in over 16s: service delivery and organisation. Nice guideline 94 ed, 2018:1-39.
8. Huby G, Harris FM, Powell AE, et al. Beyond professional boundaries: relationships and resources in health services' modernisation in England and Wales. *Sociology of Health & Illness* 2014;36(3):400-15. doi: doi:10.1111/1467-9566.12067
9. Tanabe K, Sawada K, Shimada M, et al. Evaluation of A Novel Information-Sharing Instrument for Home-Based Palliative Care. *American Journal of Hospice & Palliative Medicine* 2015;32(6):611-19. doi: 10.1177/1049909114533141
10. Powell AE, Davies HT. The struggle to improve patient care in the face of professional boundaries. *Social science & medicine* 2012;75(5):807-14.
11. Kreindler SA, Dowd DA, Dana Star N, et al. Silos and Social Identity: The Social Identity Approach as a Framework for Understanding and Overcoming Divisions in Health Care. *The Milbank Quarterly* 2012;90(2):347-74. doi: doi:10.1111/j.1468-0009.2012.00666.x
12. Braithwaite J, Clay-Williams R, Vecellio E, et al. The basis of clinical tribalism, hierarchy and stereotyping: a laboratory-controlled teamwork experiment. *BMJ Open* 2016;6(7) doi: 10.1136/bmjopen-2016-012467
13. Kirk A, Crompton PW, Knighting K, et al. Paramedics and their role in end-of-life care: perceptions and confidence. *Journal of Paramedic Practice* 2017;9(2):71-79.
14. Murphy-Jones G, Timmons S. Paramedics' experiences of end-of-life care decision making with regard to nursing home residents: an exploration of influential issues and factors. *Emergency Medicine Journal* 2016;33(10):722-6.
15. Thompson GN, Menec VH, Chochinov HM, et al. Family satisfaction with care of a dying loved one in nursing homes: what makes the difference? *Journal of Gerontological Nursing* 2008;34(12):37-44. doi: 10.3928/00989134-20081201-10
16. Street A, Blackford J. Communication issues for the interdisciplinary community palliative care team. *J CLIN NURS* 2001;10(5):643.
17. NHS England. Actions for End of Life Care: 2014-16. In: England N, ed. Leeds: NHS England, 2014.
18. NHS National Information Board. Personalised Health and care 2020: Using Data and Technology to Transform Outcomes for Patients and Citizens. In: Board NNI, ed., 2014.

For peer review only

19. NHS Improving Quality. Economic Evaluation of the Electronic Palliative Care Coordination System (EPaCCS) Early Implementer Sites, 2013.
20. Wye L, Lasseter G, Simmonds B, et al. Electronic palliative care coordinating systems (EPaCCS) may not facilitate home deaths: a mixed methods evaluation of end of life care in two English counties. *Journal of Research in Nursing* 2016;21(2):96-107.
21. Dalkin S, Lhussier M, Jones D, et al. Open communication strategies between a triad of 'experts' facilitates death in usual place of residence: A realist evaluation. *Palliative Medicine* 2018;32(5):980-89. doi: 10.1177/0269216318757132
22. NHS Improving Quality. Electronic Palliative Care Co-ordination Systems (EPaCCS) in England: Survey of clinical commissioning groups (2013) by the National End of Life Care Intelligence Network. In: England PH, ed. Public Health England, 2013.
23. Oakley A, Strange V, Bonell C, et al. Process evaluation in randomised controlled trials of complex interventions. *Bmj* 2006;332(7538):413-16.
24. May C, Finch T. Implementing, embedding, and integrating practices: an outline of normalization process theory. *Sociology* 2009;43(3):535-54.
25. Greenhalgh T, Robert G, Macfarlane F, et al. Diffusion of innovations in service organizations: systematic review and recommendations. *The Milbank Quarterly* 2004;82(4):581-629.
26. Braun V, Clarke V. Using thematic analysis in psychology. *Qualitative research in psychology* 2006;3(2):77-101.
27. England N. Lessons learned review of the WannaCry Ransomware Cyber Attack. https://www.england.nhs.uk/wp-content/uploads/2018/02/lessons-learned-review-wannacry-ransomware-cyber-attack-cio-review.pdf, 2018.
28. News B. Facebook-Cambridge Analytica scandal https://www.bbc.co.uk/news/topics/c81zyn0888lt/facebook-cambridge-analytica-scandal2019 [cited 2019 10th December]. Available from: https://www.bbc.co.uk/news/topics/c81zyn0888lt/facebook-cambridge-analytica-scandal accessed 10th December 2019.
29. Whole Systems Partnership. Independent evaluation of Electronic Palliative Care Co-ordination Systems (EPaCCS) in England, 2016.
30. Hall S, Murchie P, Campbell C, et al. Introducing an electronic Palliative Care Summary (ePCS) in Scotland: patient, carer and professional perspectives. *Family practice* 2012;29(5):576-85.
31. Patterson R, Standing H, Lee M, et al. Paramedic information needs in end-of-life care: a qualitative interview study exploring access to a shared electronic record as a potential solution. *BMC Palliative Care* 2019;18(1):108. doi: 10.1186/s12904-019-0498-2
32. Leniz J, Weil A, Higginson IJ, et al. Electronic palliative care coordination systems (EPaCCS): a systematic review. *BMJ Supportive and Palliative Care* 2019:bmjspcare-2018-001689. doi: 10.1136/bmjspcare-2018-001689
33. Dignam C, Thomas J, Brown M, et al. The impact of language on the interpretation of resuscitation clinical care plans by doctors. A mixed methods study. *PloS one* 2019;14(11)
34. Mack DS, Dosa D. Improving Advanced Care Planning through Physician Orders for Life-Sustaining Treatment (POLST) Expansion across the United States: Lessons Learned from State-Based Developments. *American Journal of Hospice and Palliative Medicine®* 2020;37(1):19-26. doi: 10.1177/1049909119851511

For peer review only

Funding statement

This paper summaries independent research funded by Connected Health Cities (CHC). The views expressed are those of the authors and not necessarily those of CHC.

Competing interests

We have no competing interests to declare.

Authors Contributions

HS, RP, ML, SD, MLh, AB, CE and KB (all authors) contributed to the design of the study. HS KB and RP conducted the data collection and analysis. HS and KB drafted the manuscript. All authors revised the article critically for important intellectual content. All authors approved the submitted draft.

Acknowledgements

We acknowledge the funder, the wider project team (led by Dr Kathryn Hall, clinical lead), Michele Spencer from North Tyneside Community and Health Care Forum and most importantly the participants who gave their time to the research.

Data Availability Statement

Given the sensitive nature of this qualitative research project the raw data generated and analysed during the current study are not publicly available to preserve the anonymity of participants. However, we are willing to make portions of transcripts (with any identifying information redacted) available upon reasonable request. Interested persons should contact the corresponding author.

For peer review only

No	Item	Guide questions/description	Answer
**Domain 1: Research team and reflexivity**			
Personal Characteristics			
1.	Interviewer/facilitator	Which author/s conducted the interview or focus group?	Holly Standing, Rebecca Patterson, and Katie Brittain conducted the interviews
2.	Credentials	What were the researcher's credentials? *E.g. PhD, MD*	Holly Standing, PhD, MSc, BSc<br>Rebecca Patterson – MSc, PGCert, BA (hons)<br>Katie Brittain - PhD, MA, BSc (hons)
3.	Occupation	What was their occupation at the time of the study?	Holly Standing- Vice Chancellor's Research Fellow<br>Rebecca Patterson – Senior Research Assistant<br>Katie Brittain – Associate Professor of Ageing & Health
4.	Gender	Was the researcher male or female?	Female
5.	Experience and training	What experience or training did the researcher have?	All have significant experience of conducting interviews and focus groups

For peer review only

No	Item	Guide questions/description	Answer
Relationship with participants			
6.	Relationship established	Was a relationship established prior to study commencement?	Participants were known to the researcher via emails/phone calls prior to interview.
7.	Participant knowledge of the interviewer	What did the participants know about the researcher? e.g. *personal goals, reasons for doing the research*	The participants had been provided with an information sheet about the research project and given the opportunity to ask the researchers any questions prior to interview.
8.	Interviewer characteristics	What characteristics were reported about the interviewer/facilitator? e.g. *Bias, assumptions, reasons and interests in the research topic*	None
**Domain 2: study design**			
Theoretical framework			
9.	Methodological orientation and Theory	What methodological orientation was stated to underpin the study? *e.g. grounded theory, discourse analysis, ethnography, phenomenology, content analysis*	Explorative qualitative study, thematic approach to analysis

For peer review only

No	Item	Guide questions/description	Answer
Participant selection			
10.	Sampling	How were participants selected? *e.g. purposive, convenience, consecutive, snowball*	Opportunistic sampling
11.	Method of approach	How were participants approached? e.g. *face-to-face, telephone, mail, email*	Via email
12.	Sample size	How many participants were in the study?	71
13.	Non-participation	How many people refused to participate or dropped out? Reasons?	1 expressed an interest but did not arrange an interview. No specific reason was given.
Setting			
14.	Setting of data collection	Where was the data collected? e.g. *home, clinic, workplace*	University, public spaces (cafes), interviewees workplace, via the telephone
15.	Presence of non-participants	Was anyone else present besides the participants and researchers?	No

For peer review only

No	Item	Guide questions/description	Answer
16.	Description of sample	What are the important characteristics of the sample? *e.g. demographic data, date*	62 health and social care professionals (GP, out-of-hours GPs, nurses, formal carers, paramedics, social workers, pharmacists, hospital doctors, other supporting professionals), 9 patients and family members (6 female, 3 male, 1 <60, 8 60+)
Data collection			
17.	Interview guide	Were questions, prompts, guides provided by the authors? Was it pilot tested?	Topic guides were used to structure the interviews, these were a living documents which were expanded throughout data collection to explore issues raised in earlier interviews.
18.	Repeat interviews	Were repeat interviews carried out? If yes, how many?	No
19.	Audio/visual recording	Did the research use audio or visual recording to collect the data?	Audio recording was used in all interviews
20.	Field notes	Were field notes made during and/or after the interview or focus group?	Relevant notes were made during and after the interview
21.	Duration	What was the duration of the interviews or focus group?	Interviews between 22 and 93 mins.
22.	Data saturation	Was data saturation discussed?	No

For peer review only

No	Item	Guide questions/description	Answer
23.	Transcripts returned	Were transcripts returned to participants for comment and/or correction?	No
**Domain 3: analysis and findings**			
Data analysis			
24.	Number of data coders	How many data coders coded the data?	1 main coder (RP), reviewed by an expert group of 4 individuals (KB, HS, ML, SD)
25.	Description of the coding tree	Did authors provide a description of the coding tree?	No
26.	Derivation of themes	Were themes identified in advance or derived from the data?	Derived from the data
27.	Software	What software, if applicable, was used to manage the data?	Yes – Nvivo 12
28.	Participant checking	Did participants provide feedback on the findings?	No
Reporting			

For peer review only

No	Item	Guide questions/description	Answer
29.	Quotations presented	Were participant quotations presented to illustrate the themes / findings? Was each quotation identified? e.*g. participant number*	Yes
30.	Data and findings consistent	Was there consistency between the data presented and the findings?	Yes
31.	Clarity of major themes	Were major themes clearly presented in the findings?	Yes
32.	Clarity of minor themes	Is there a description of diverse cases or discussion of minor themes?	No

For peer review only

BMJ Open

Information sharing challenges in end-of-life care: a qualitative study of patient, family and professional perspectives on the potential of an EPaCCS in the North East of England

Journal:	*BMJ Open*
Manuscript ID	bmjopen-2020-037483.R1
Article Type:	Original research
Date Submitted by the Author:	29-May-2020
Complete List of Authors:	Standing, Holly ; Northumbria University, Department of Nursing Midwifery and Health<br>Patterson, Rebecca; Northumbria University, Research and Innovation Services<br>Lee, Mark; St Benedict's Hopsice and Specialist Palliative Care Centre<br>Dalkin, Sonia; Northumbria University Faculty of Health and Life Sciences, School of Health Education and Community Studies<br>Lhussier, Monique; Northumbria University Faculty of Health and Life Sciences, School of Health, Education and Community Studies<br>Bate, Angela; Northumbria University, Nursing, Midwifery and Health<br>Exley, Catherine; Newcastle University, Population Health Sciences Institute<br>Brittain, Katie; Newcastle University, Population Health Sciences Institute
**Primary Subject Heading**:	Qualitative research
Secondary Subject Heading:	Palliative care
Keywords:	Organisation of health services < HEALTH SERVICES ADMINISTRATION & MANAGEMENT, PALLIATIVE CARE, QUALITATIVE RESEARCH, Information technology < BIOTECHNOLOGY & BIOINFORMATICS

SCHOLARONE™
Manuscripts

I, the Submitting Author has the right to grant and does grant on behalf of all authors of the Work (as defined in the below author licence), an exclusive licence and/or a non-exclusive licence for contributions from authors who are: i) UK Crown employees; ii) where BMJ has agreed a CC-BY licence shall apply, and/or iii) in accordance with the terms applicable for US Federal Government officers or employees acting as part of their official duties; on a worldwide, perpetual, irrevocable, royalty-free basis to BMJ Publishing Group Ltd ("BMJ") its licensees and where the relevant Journal is co-owned by BMJ to the co-owners of the Journal, to publish the Work in this journal and any other BMJ products and to exploit all rights, as set out in our licence.

The Submitting Author accepts and understands that any supply made under these terms is made by BMJ to the Submitting Author unless you are acting as an employee on behalf of your employer or a postgraduate student of an affiliated institution which is paying any applicable article publishing charge ("APC") for Open Access articles. Where the Submitting Author wishes to make the Work available on an Open Access basis (and intends to pay the relevant APC), the terms of reuse of such Open Access shall be governed by a Creative Commons licence – details of these licences and which Creative Commons licence will apply to this Work are set out in our licence referred to above.

Other than as permitted in any relevant BMJ Author's Self Archiving Policies, I confirm this Work has not been accepted for publication elsewhere, is not being considered for publication elsewhere and does not duplicate material already published. I confirm all authors consent to publication of this Work and authorise the granting of this licence.

For peer review only

Information sharing challenges in end-of-life care: a qualitative study of patient, family and professional perspectives on the potential of an EPaCCS in the North East of England

Holly C Standing[1], Rebecca Patterson[2], Mark Lee[3], Sonia Michelle Dalkin[4], Monique Lhussier[4], Angela Bate[1], Catherine Exley[5], and Katie Brittain[5]

[1]Department of Nursing, Midwifery and Health, Faculty of Health and Life Sciences, Northumbria University

[2]Research and Innovation Services, Northumbria University

[3]St Benedict's Hospice and Specialist Palliative Care Centre, Sunderland

[4]Department of Social Work, Education and Community Wellbeing, Faculty of Health and Life Sciences, Northumbria University

[5]Population Health Sciences Institute, Faculty of Medical Sciences, Newcastle University

Corresponding author: Dr Holly C Standing, Northumbria University, Coach Lane Campus West, Newcastle Upon Tyne, NE77AX (holly.standing@northumbria.ac.uk) 0191 232 6002

Word count 4,631

Abstract

Objectives To explore current challenges in inter-disciplinary management of end-of-life care in the community, and the potential of an Electronic Palliative Care Coordination System (EPaCCS) to facilitate the delivery of care which meets patient preferences.

Design Qualitative study using interviews and focus-groups.

Setting Health and Social Care Services in the North of England.

Participants 71 participants, 62 health and social care professionals, 9 patients and family members.

For peer review only

Results Four key themes were identified: information sharing challenges; information sharing systems; perceived benefits of an EPaCCS; barriers to use and requirements for an EPaCCS. Challenges in sharing information were a source of frustration for health and social care professionals as well as patients, and were suggested to result in inappropriate hospital admissions. Current systems were perceived by participants to not work well - paper advance care planning documentation was often unavailable or inaccessible, meaning it could not be used to inform decision-making at the point of care. Participants acknowledged the benefits of an EPaCCS to facilitate information sharing, however they also raised concerns about confidentiality, and availability of the increased time and resources required to access and maintain such a system.

Conclusions EPaCCS offer a potential solution to information sharing challenges in end-of-life care. However, our findings suggest that there are issues in the initiation and documentation of end-of-life discussions that must be addressed through investment in training in order to ensure that there is sufficient information regarding ACP to populate the system. There is a need for further qualitative research evaluating use of an EPaCCS, which explores benefits and challenges, uptake and reasons for disparities in use to better understand the potential utility and implications of such systems.

Keywords

Information technology, organisation of health services, palliative care, qualitative research

ARTICLE SUMMARY

Strengths and limitations of this study

For peer review only

- Large participant group from various stakeholder groups including health and social care practitioners, patients and carers
- This is the first study to include the experience of social workers and of a coroner
- Semi-structured interviews allowed us to collect rich descriptions of participants' experiences and perceptions of information sharing in end-of-life care and of an EPaCCS
- The study was conducted by a team of experienced researchers from a range of disciplinary backgrounds
- Data are from the pre-implementation phase of an EPaCCS trial, so findings are not drawn from direct experience of the system.

INTRODUCTION

The development of end-of-life care guidance in recent years supports attempts to allow dying persons more control over their end-of-life experience, through engagement of patients in Advance Care Planning (ACP).[1] ACP has been defined as 'a process that supports adults at any age or stage of health in understanding and sharing their personal values, life goals, and preferences regarding future medical care'. The goal of advance care planning is to help ensure that people receive medical care that is consistent with their values, goals and preferences during serious and chronic illness.'[2] ACP can include anyone involved in the provision of the patient's care, including nurses, doctors, care home managers and family members. Documents completed as part of ACP can include: a do not attempt cardiopulmonary resuscitation order (DNACPR), emergency health care plan (EHCP), and a written summary of the patients care preferences.[3] Paper versions of this documentation are completed and held by the patient to present to the health and social care professionals they

For peer review only

encounter.[4] ACP can facilitate the delivery of care which better reflects the patient's personal preferences,[1] and can have positive impacts on the person at the end-of-life and their family.[5] However, evidence suggests that the completion and use of ACP documentation is inconsistent.[6 7]

The delivery of high quality end-of-life care is an inter-professional endeavour, where the resources of various health and social care professionals must be combined for the benefit of the patient.[8] To achieve this, efficient inter-professional communication and information sharing across health and social care professionals involved in community end-of-life care is essential[9], but presents many challenges.[10-12] Accessibility of patient information can be limited, particularly for services who may only be episodically involved in end-of-life care but that offer crucial and timely support, such as paramedics, out-of-hours doctors, Admiral nurses (dementia specialist nurses), care home staff, and social workers.[13 14] Poor (or lack of) access to patient information can effect professionals' confidence in managing end-of-life patients, [13 14] family members satisfaction with care, [15] and patient outcomes.[5] Sharing of patient information is made more challenging by inconsistent documentation practices and systems which vary across organisations and professional boundaries.[16]

Electronic record systems offer a potential solution to these information sharing challenges.[17] A Framework for Action published by the National Information Board in 2014, details 2020 as a deadline for digital, real-time, and interoperable health records to be rolled out within the NHS.[18] Electronic Palliative care Co-ordinating Systems (EPaCCS) are an example of such an innovation. EPaCCS is an umbrella term covering a number of different electronic solutions that aim to capture patient wishes and preferred place of death and improve co-ordination of care in real time, through enabling the sharing of information across health and social care services. A 2013 Department of Health pilot evaluation in England estimated that EPaCCS could save £133,200 in hospital admissions per annum per 200,000 patient

For peer review only

population.[19] There are a number of versions of EPaCCS currently in use in the UK including the Key Information Summary (KIS) in Scotland,[20] Coordinate My Care in London,[21] South West EPaCCS[22] and Leeds EPaCCS[23]. Like many areas of the UK, the North East England does not yet have an EPaCCS in place. Research from other regions indicates that professional uptake and patient registration on such electronic systems is currently variable.[20 22] A systematic review found that 55% to 79% of patients with an EPaCCS died in their preferred place, higher than the general population.[24] The Scottish KIS has been suggested to be particularly beneficial for GPs working out of hours.[25] Challenges surrounding the use of an EPaCCS include professional reluctance to initiate end-of-life discussions,[6 26] increased burden from data entry for the primary users,[22] data ownership, funding, and consent.[27]

The aim of our study was to explore attitudes towards the potential of an EPaCCS solution for improving information sharing and coordination in end-of-life care within the North East of England. We provide an in-depth study of the professional and organisational factors that promote or inhibit the acceptability, usefulness and integration of collaborative care planning across health and social care into service delivery and everyday practice.[28-30]

METHODS

This study employed qualitative methods, involving semi-structured interviews and focus groups with patients, bereaved family members, and health and social care professionals.

Participants

A heterogeneous purposive sampling approach was employed to ensure representation from all health and social care professional groups who have a potential role in end-of-life care, and that a variety of perspectives and experiences of end-of-life care were sampled. Ethical approval for this study was obtained from the Health Research Authority (REC reference: 17/LO/2100).

For peer review only

Data collection

An interview schedule was developed by HS, RP and KB which included: current approaches to end-of-life care management, access to patient data, and attitudes towards introduction of an EPaCCS (including perceived benefits and challenges) an example interview schedule is provided in the supplementary material. A video demonstration of a draft version of the proposed EPaCCS was also used as a prompt during clinician interviews. The interview schedule evolved during the data collection process to allow iterative data analysis and further exploration of themes derived from the data. Informed consent was given by all participants prior to taking part. Interviews were digitally recorded.

Participants were recruited from an area within the North East of England where an EPaCCS was being trialled, data presented are from the pre-implementation phase of the trial. Patient and family participants were recruited through GP practices, practice staff acted as gatekeepers and sent information packs to patients on their palliative care registers and next of kin of deceased patients, those interested in participating returned a consent-to-contact form in a prepaid envelope. Clinicians were recruited through local clinical networks, calls for volunteers circulated via email, attendance of the study team at local commissioning meetings.   Data collection took place from March to October 2018, interviews and focus groups were conducted at the participants' place of work or home, at university or community venues, or over the telephone, according to participant preference. Interviews and focus groups were conducted by HS, RP and KB. Clinician data were generated through 24 interviews and 5 focus groups. Two of the five focus groups were conducted with a mix of health and social care professionals including hospital doctors, pharmacists, out-of-hours GPs (n=9 and n=10). The remaining three focus groups were conducted with single profession groups: care home staff (n=5), social workers (n=5), nurse (n=9). Cancer was the primary diagnosis for all patient interviews and family interviews. Table 1 details participant

For peer review only

demographics. Clinician interviews ranged from 28-93 minutes, focus groups from 39-63 minutes, patients and family member interviews from 22 and 80 minutes. Field notes were collected before, during and after data collection. Data has been pseudonymised to protect participant identity.

Table 1: Participant demographics

**Health and social care professional role**	**No.**
GP	7
Out of hours GP	2
Nurse (including: Macmillan [cancer care nurse], Marie Curie [terminal care nurse], care home, district, rapid response, Admiral nurse [dementia care nurse])	21
Formal carer (care home)	3
Paramedic	10
Social worker	6
Pharmacist	4
Hospital doctor	4
Other supporting professions (including: care coordinator, Macmillan manager, physiotherapist, coroner, palliative care team)	5
**Patient/family**	
Patient	7 (4 female, 5 lived alone, 6 60+ years old)
Family	2 (both female, 60+)
**Total**	**71**

Data Analysis

Audio recordings were transcribed verbatim by an independent transcription company; transcripts were checked for accuracy by (HS, RP, KB). The data management software NVivo 12 was used to develop and refine a coding scheme.

For peer review only

We adopted an iterative approach to data analysis. Data collection and analysis ran concurrently throughout the study and analysis of early transcripts informed the refinement of the interview schedule for later interviews. The five-step process of thematic analysis [31] was adopted to develop and refine codes and themes from the data. Field notes taken during data collection were utilised in data analysis to enhance the reflexive process. Credibility measures included a process of continuous review and discussion of themes at group analysis sessions, and at wider team meetings. Three researchers (HS, RP, KB) independently coded 20% of the transcripts before coming together to compare codes and discuss discrepancies.

Patient and public involvement

The study was supported by a patient and public involvement representative who contributed to the design and development of the study, advised on recruitment processes, and participant information and consent materials.

FINDINGS

Four themes were identified through data analysis: information sharing challenges; information sharing systems; perceived benefits of an EPaCCS; barriers to use and requirements for an EPaCCS. These are presented in turn below, with illustrative quotations.

Information sharing challenges

Participants were keen for end-of-life patients in the community to be managed at home and avoid inappropriate hospital admissions where possible, however, poor communication and information sharing between services was a barrier to achieving this goal. Paramedics and out-of-hours GPs may be alerted by call handlers that the patient they are attending is at the end-of-life, but often were going in 'blind' as they had no prior access to the patient's condition or care preferences. Without access to up-to-date patient information, paramedics felt that in such situations they had no choice but to admit the patient:

For peer review only

The number of times I've said, "They are going to have to go in. I don't want to take them in, but I don't have a choice here, because I don't have the information." If you don't have the information, you can't make an informed decision, so to leave them at home would be the wrong thing to do, even though you know morally you'd like to. (Paramedic6)

Inappropriate hospital admission of end-of-life patients was also a source of frustration for GPs who suggested that these could be avoided if they were contacted for advice:

I suppose the admissions where they're palliative are the ones that I get annoyed about [...] they get admitted, and if somebody had asked you, you know, "What should we do with this lady?" [we could advise on management but]*that's not always easy because we don't work every day. We are obviously not working in the evenings or the weekends*[...] (GP6)

Indeed, the continuity of care afforded by the GP role means they often have established relationships with end-of-life patients and would most likely have access to detailed information regarding their condition, history and care preferences, which should inform decision making in any circumstance. However as in-hours GPs are not available 24/7 there is a need for patient information to be stored in a manner that is easily accessible to all services who may require it. Paramedics or out-of hours GPs are unlikely to have had prior contact when called out to an end-of-life patient, and would benefit to access to greater patient information to facilitate their management of such patients, and potentially avoid hospital admissions.

Poor communication and information sharing could have negative impacts on the patient and

For peer review only

their family. Where patient information is unavailable, and services were going in 'blind' the burden of information sharing falls on the patient or their family.

> *Whatever doctor I would see, they wouldn't know a lot about me, so I'd have to constantly start from the beginning* […family] *couldn't come into the meetings with me because they'd get so upset because they'd watch me explain everything from the start. I think that was worse than hearing any bad news* (Patient5)

Having to repeatedly reiterate their story could be distressing for the patient and their family, and could also damage the professional patient relationship; as the patient above describes this constant repeating of her story led her family to disengage from encounters with health and social care professionals.

Lack of information sharing mean it is not always clear who should be responsible for verifying death and issuing the death certificate. This could result in the death being designated as 'unexpected' and the coroners being inappropriately involved, causing unnecessary delays and distress to the family:

> *when they've gone home expecting that* [the patient will die] *and then all of a sudden somebody says, "We don't know who can issue the certificate because the GP hasn't seen them because it was the out of hours doctor* [who came]…" *and it takes hours to sort it out*. (Coroner1)

Information sharing systems

Information about patients at the end-of-life is primarily stored in two formats: 1) paper-based ACP documents which are held by the patient, and 2) isolated patient record systems

For peer review only

(as it currently stands) versus an integrated system that can be updated and available to all professionals (and patients in 'real time').

Paper ACP documents facilitate information sharing, however, their usefulness is largely dependent on the patient, or their family, having the documents ready to hand or easily accessible and up to date. Many participants indicated this was often not the case:

> *you're meant to have a bright yellow envelope on your mantle or on your sideboard. So, if you go in as a doctor, or if you go in as an ambulance crew, you know it's there, and it's bright, illuminous yellow, but the number of folks, "Oh, I've got one of those," and it's under four piles of stuff, or it's in their bedside cabinet. It's like, "Well, no-one's going to respect that," because no-one's going to spend 10 minutes looking for it if you've arrested. They're going to start resus.* (Out-of-hours GP1)

If the documentation is not available, either misplaced or forgotten, then health and social care professionals have to proceed as if it did not exist.

Having a standardised location for the documentation in the patient's home (such as the mantelpiece, for example) may improve access to this information. However, keeping the documentation in a visible location may also be a potential source of distress for families. Whilst some patient participants were comfortable having theirs visible, others chose to keep them hidden as they acted as a constant reminder of the imminent death.

> *I had put the yellow envelope in my knicker drawer* […] *They said, "Put it up there."* [mantel piece] *So, I said, "No, I'm not putting it up there. I don't want to look at that all the time."* (Bereaved family2)

For peer review only

In addition to paper versions of ACP documentation where available, health and social care services utilise electronic patient record systems to record and manage the information that they collect. These systems tend to work in isolation, and are generally not inter-operable, meaning there is no simple way to share information with others. Many patients assumed that their information was being shared, and they did not necessarily perceive services as discrete organisations, rather part of a united healthcare system. Although patients recognised that protecting patient confidentially was important, the benefits of sharing information were perceived to significantly outweigh these concerns.

> *What if you travel? What if you're visiting round the country? You don't know what's going to happen* […] *I went to London a couple of times when I was ill. What happens if something happens to me down there? How long would it take them to find anything out about me? In the meantime, what's happening to me?* (Patient5)

Perceived benefits of an EPaCCS

Participants identified several potential benefits of adopting an EPaCCS. NHS electronic patient records systems were perceived by the majority of participants, both patient and professional, as being extremely disjointed and often outdated.

> *I certainly think there should be a national set-up. Everybody should be reading from the same sheet, yes.* (Patient6)

Patients welcomed a system that would facilitate better transfer of information between services so that they would no longer have to act as the conduit for passing information between services by repeating their story.

For peer review only

Professionals indicated that an EPaCCS may save time and facilitate the provision of care that was better aligned with patients' wishes. An EPaCCS could potentially allow multiple users to simultaneously access and update a patient's record, ensuring changes to the patient's condition and/or care plan are immediately accessible to all professionals involved in their care.

> *I think the thing is, when it's a paper copy there's only one actual current live copy that is up to date* […] *the electronic copy means multiple users could be using it at the same time.* (Nurse6)

An EPaCCS was suggested to be particularly beneficial to services without sustained relationships with end-of-life patients who were often 'information poor'. Improved access to patient information could potentially increase the confidence of these services in delivering end-of-life care.

> *You do get very limited information and you're going into a person to talk to them about what care they need with very limited information. And sometimes when they are end-of-life, the last thing they want to talk about is how they're going to die and why they're going to die* (Social Worker5)

Barriers to use, and requirements for an EPaCCS

Professional participants did not express universal enthusiasm for the introduction of an EPaCCS. Paramedics and out-of-hours GPs were generally positive about the potential of the system, as discussed above, however GPs were less enthusiastic; the system was not necessarily perceived to offer improvements over current approaches to information storing and recording. GPs expressed concern about implications of the system for their workload; GPs are often the most 'information rich' meaning responsibility for inputting data into the system would fall on them. If the proposed EPaCCS was not fully inter-operable with

For peer review only

existing systems it would result in duplication of tasks and increased demands on GP time.

> If it's [a] *separate* [system] *then you'd be duplicating because you'd need to add it to the system wouldn't you?* […] *You'd find GPs wouldn't engage with it, because it would just be an extra thing they have to do.* (GP3)

The implications of a shared EPaCCS on maintaining data protection and the security of patient information was a major concern for many of the professional participants.

> *We're still recovering from a cyber-attack a year ago, and there are still some documents that we can't open* […] *so it has to be encrypted to a level that it's safe* (Hospital doctor3)

Such concerns are understandable given recent security threats [32] and scandals involving misuse of personal data.[33] However, this did not appear to be a concern for patients, many of whom assumed that data sharing practices already occurred and viewed moves to facilitate information sharing through electronic records with the aim of improving care as welcome and necessary progress.

Incorporating the informational requirements of all health and social care groups into one system was highlighted as a challenge; different roles have different informational needs. For example, nurses indicated that lists of patient medication are essential information for their practice to ensure they are not prescribing contraindicated medications. Catering for individualised requirements such as these risks the EPaCCS becoming overwhelming and challenging to navigate.

For peer review only

It might just slow people down. It will take longer actually to get through cases because you have to read through them [patient notes] (Out-of-hours GP1)

Having to sift through irrelevant information to find what is germane to their role may become burdensome. An EPaCCS with extensive information about the patient and limited in its ability to filter efficiently may not be preferable to paper ACP documentation being available upon arrival.

Professionals who spend most of their time working remotely, doing home visits, may gain the most benefit from an EPaCCS.

electronic information, that would solve the main problems that we have, which is lack of information at times. So yes, that would be great. (Paramedic5)

However, an EPaCCS needs to be accessible to these professionals. If an EPaCCS cannot be accessed and updated by those working in the community, the information contained within will quickly cease to be up-to-date. Successful uptake of an EPaCCS is also dependent upon the technology available to access the system. Electronic devices available to those working in the community were reported to often be of low specification, raising doubts about likelihood that they would be able to support accessibility.

It also has to be an 'on the go' portal, you were saying how busy you are and so it has to work on a tablet or on an iPad because a lot of this work is being inputted in the community. You can't rely on someone going back to their office, after they've done five house calls or five social work visits or

For peer review only

five whatever [..] *It has to be a real-time and easy to access.* (Hospital Doctor2)

A need for ongoing support for the EPaCCS following the implementation period was highlighted. Participants expressed concern that investing time learning a new system would be wasted if ongoing infrastructure was not available to support its continued use.

I've done many projects before within health, and there's a massive emphasis placed at the beginning of every project and they put it on. Then, afterwards, staff move away, because it's not part of their project and we're suddenly left with it. The system is changing and evolving, but there's not always the infrastructure in place to support us to update it. I think that's a real concern. (Nurse19)

Implementation of an EPaCCS was not necessarily perceived to solve the challenges in inter-disciplinary management of end-of-life care alone. Access to ACP documentation will only be useful if the documentation itself is completed to a satisfactory standard, if insufficient detail is provided it cannot be used to inform decision making. Paramedics and out-of-hours doctors indicated that ACP documentation that they encountered was of a variable standard, and these documents should be completed with these services in mind as the intended reader.

I'm making a decision within 30 seconds I've walked through the door, "Am I going to attempt to save a life or am I going to let them expire?" The only thing between me and setting off a treatment path is the end-of-life care plan, so it needs to make perfect sense.(Paramedic4)

For peer review only

DISCUSSION

Principle statement of findings

This study provides insight into current challenges in information sharing in end-of-life care, which were recognised as a barrier to effective end-of-life care, and the potential of an EPaCCS system to overcome these challenges. Inconsistent information sharing within and between health and social care services was a source of frustration for patients, paper based ACP documentation does not appear to be an effective system for recording and sharing patients' care preferences; often these documents are unavailable when required and are variable in quality. Patients welcomed the idea of an EPaCCS that facilitated sharing of their information between health and social care services, they did not report the same concerns about data protection and security issues that concerned clinicians. Both groups of participants perceived the potential for improvements in delivery aligned with patient's wishes from adoption of an EPaCCS. Perceived barriers to the successful implementation of an EPaCCS were focused on increased demand on time and the lack of infrastructure in place to support the system. Care must be taken to ensure that information contained within the EPaCCS does not become overwhelming. This is particularly important for emergency services, such as paramedics, who work under extreme time pressures limiting their ability to access and digest vast amounts of information. Introduction of an EPaCCS was not seen to resolve current challenges in management of patients at the end-of-life without investment into improving the frequency with which ACP documentation is completed and the quality of the information recorded. Paramedics in particular suggested their informational needs must be a priority to ensure ACP documentation is completed to a standard where it can be used to inform decision making.

For peer review only

Strengths and weaknesses

A key strength of this study is the large sample which includes a wide range of health and social care professionals involved in end-of-life care, the majority of previous qualitative studies have employed small samples,[34 35] and ours is the first study to our knowledge to include the perspectives of supporting professions such as social workers and coroners. In addition, our study included a greater number of participants from out-of-hours services than previous qualitative studies.[22 35] As the data were collected during the developmental pre-implementation phase of an EPaCCS trial, participants were only able to reflect hypothetically on the impacts of such a system on their practice rather than drawing on direct experiences of working with the system. In line with previous EPaCCS studies, a second limitation of the study stems from the recruitment process for patients and family members, which was, operationalised through GP practices. Staff thus acted as gatekeepers and may have been selective in who they referred for participation.

Comparison with other work

There are only a limited number of qualitative studies exploring attitudes towards and use of EPaCCS.[22 34-36] Our findings support those of a recent systematic review which identified the burden of inputting data and IT systems as the main challenge to implementation of EPaCCS.[24] GP participants expressed a reluctance to dedicate time to inputting data into the EPaCCS, this has been observed in previous studies where GPs have reported delegating much of this work to administrative staff.[22] However, there is high use of the Scottish KIS system despite reliance on GPs to populate.[20]

A key finding of our study was that introduction of an EPaCCS alone does not provide a solution to some of the current difficulties regarding inter-disciplinary management of end-of-life patients in the community. An EPaCCS will only facilitate access to patient

For peer review only

information, the utility of which is largely dependent upon its quality; improving access to ACP documentation is not going to improve care if the information recorded within them is of poor quality or insufficient detail, or has not been completed. This demonstrates a need for investment in training for health and social care professionals in ACP discussions and documentation to instil confidence and improve quality. Inconsistencies in ACP documentation have also been observed within an Australian healthcare context.[37]

This article highlights concerns regarding the infrastructure required to support an EPaCCS including: demand on already stretched professionals, technology provision required to enable access, and continued support to ensure the system is updated. Ensuring appropriate infrastructure is in place has emerged as a challenge in the implementation of coordinated records for end-of-life patients in both UK and US contexts.[24 38] As this article focuses on the pre-implementation stage of an EPaCCS trial future research should explore the long-term uptake and impact of an EPaCCS to better understand the infrastructure required to stain such a system. Although there have been recent mixed methods studies conducted post-implementation of an EPaCCS,[20 25 39] there is a need for further qualitative research exploring the benefits and challenges experienced whilst using the system for all stakeholders including in-hours and out-of-hours services, and in particular on the impacts of the experiences of patients and families. High and low users of the system should be explored to identify reasons for disparities and potential ways of promoting use of the system.

Conclusions

EPaCCS may offer a potential solution to information sharing difficulties in the inter-disciplinary management of end-of-life care. However, implementation of an EPaCCS alone will not improve management of patients at the end-of-life it will only facilitate the access to patient information. Introduction of EPaCCS must be accompanied by investment in training

For peer review only

around communication skills focusing on the initiation of end-of-life discussions and effective completion of ACP documentation.

REFERENCES

1. NICE. Advance Care Planning: Emergency and acute medical care in over 16s: service delivery and organisation. 2018
2. Sudore RL, Lum HD, You JJ, et al. Defining Advance Care Planning for Adults: A Consensus Definition From a Multidisciplinary Delphi Panel. *J Pain Symptom Manage* 2017;53(5):821-32.e1. doi: 10.1016/j.jpainsymman.2016.12.331 [published Online First: 2017/01/08]
3. Northern Cancer Alliance. Deciding right Regional forms https://www.northerncanceralliance.nhs.uk/deciding-right/deciding-right-regional-forms/2019 [cited 2019 6th November]. Available from: https://www.northerncanceralliance.nhs.uk/deciding-right/deciding-right-regional-forms/ accessed 6th November 2019.
4. Mullick A, Martin J, Sallnow L. An introduction to advance care planning in practice. *Bmj* 2013;347:f6064.
5. Detering KM, Hancock AD, Reade MC, et al. The impact of advance care planning on end of life care in elderly patients: randomised controlled trial. *BMJ* 2010;340 doi: 10.1136/bmj.c1345
6. Dalkin SM, Lhussier M, Philipson P, et al. Reducing inequalities in care for patients with non-malignant diseases: Insights from a realist evaluation of an integrated palliative care pathway. *Palliative Medicine* 2016;30(7):690-97. doi: 10.1177/0269216315626352
7. National Institute for Health and Care Excellence. Chapter 15 Advance Care Planning. Emergency and acute medical care in over 16s: service delivery and organisation. Nice guideline 94 ed, 2018:1-39.
8. Huby G, Harris FM, Powell AE, et al. Beyond professional boundaries: relationships and resources in health services' modernisation in England and Wales. *Sociology of Health & Illness* 2014;36(3):400-15. doi: doi:10.1111/1467-9566.12067
9. Tanabe K, Sawada K, Shimada M, et al. Evaluation of A Novel Information-Sharing Instrument for Home-Based Palliative Care. *American Journal of Hospice & Palliative Medicine* 2015;32(6):611-19. doi: 10.1177/1049909114533141
10. Powell AE, Davies HT. The struggle to improve patient care in the face of professional boundaries. *Social science & medicine* 2012;75(5):807-14.
11. Kreindler SA, Dowd DA, Dana Star N, et al. Silos and Social Identity: The Social Identity Approach as a Framework for Understanding and Overcoming Divisions in Health Care. *The Milbank Quarterly* 2012;90(2):347-74. doi: doi:10.1111/j.1468-0009.2012.00666.x
12. Braithwaite J, Clay-Williams R, Vecellio E, et al. The basis of clinical tribalism, hierarchy and stereotyping: a laboratory-controlled teamwork experiment. *BMJ Open* 2016;6(7) doi: 10.1136/bmjopen-2016-012467
13. Kirk A, Crompton PW, Knighting K, et al. Paramedics and their role in end-of-life care: perceptions and confidence. *Journal of Paramedic Practice* 2017;9(2):71-79.
14. Murphy-Jones G, Timmons S. Paramedics' experiences of end-of-life care decision making with regard to nursing home residents: an exploration of influential issues and factors. *Emergency Medicine Journal* 2016;33(10):722-6.
15. Thompson GN, Menec VH, Chochinov HM, et al. Family satisfaction with care of a dying loved one in nursing homes: what makes the difference? *Journal of Gerontological Nursing* 2008;34(12):37-44. doi: 10.3928/00989134-20081201-10

For peer review only

16. Street A, Blackford J. Communication issues for the interdisciplinary community palliative care team. *J CLIN NURS* 2001;10(5):643.
17. NHS England. Actions for End of Life Care: 2014-16. In: England N, ed. Leeds: NHS England, 2014.
18. NHS National Information Board. Personalised Health and care 2020: Using Data and Technology to Transform Outcomes for Patients and Citizens. In: Board NNI, ed., 2014.
19. NHS Improving Quality. Economic Evaluation of the Electronic Palliative Care Coordination System (EPaCCS) Early Implementer Sites, 2013.
20. Finucane AM, Davydaitis D, Horseman Z, et al. Electronic care coordination systems for people with advanced progressive illness: a mixed-methods evaluation in Scottish primary care. *British Journal of General Practice* 2019:bjgp19X707117. doi: 10.3399/bjgp19X707117
21. Smith C, Hough L, Cheung C-C, et al. Coordinate My Care: a clinical service that coordinates care, giving patients choice and improving quality of life. *BMJ supportive & palliative care* 2012;2(4):301-07.
22. Wye L, Lasseter G, Simmonds B, et al. Electronic palliative care coordinating systems (EPaCCS) may not facilitate home deaths: A mixed methods evaluation of end of life care in two English counties. *Journal of Research in Nursing* 2016;21(2):96-107. doi: 10.1177/1744987116628922
23. Henry K, Kite S, McDermott S, et al. THE DEVELOPMENT OF AN ELECTRONIC REPORTING MECHANISM TO SUPPORT THE USE OF EPACCS IN CLINICAL PRACTICE. *BMJ Supportive & Palliative Care* 2014;4(Suppl 1):A65-A66. doi: 10.1136/bmjspcare-2014-000654.186
24. Leniz J, Weil A, Higginson IJ, et al. Electronic palliative care coordination systems (EPaCCS): a systematic review. *BMJ Supportive and Palliative Care* 2019:bmjspcare-2018-001689. doi: 10.1136/bmjspcare-2018-001689
25. Tapsfield J, Hall C, Lunan C, et al. Many people in Scotland now benefit from anticipatory care before they die: an after death analysis and interviews with general practitioners. *BMJ Support Palliat Care* 2019;9(4):e28. doi: 10.1136/bmjspcare-2015-001014 [published Online First: 2016/04/15]
26. Dalkin S, Lhussier M, Jones D, et al. Open communication strategies between a triad of ‘experts’ facilitates death in usual place of residence: A realist evaluation. *Palliative Medicine* 2018;32(5):980-89. doi: 10.1177/0269216318757132
27. NHS Improving Quality. Electronic Palliative Care Co-ordination Systems (EPaCCS) in England: Survey of clinical commissioning groups (2013) by the National End of Life Care Intelligence Network. In: England PH, ed. Public Health England, 2013.
28. Oakley A, Strange V, Bonell C, et al. Process evaluation in randomised controlled trials of complex interventions. *Bmj* 2006;332(7538):413-16.
29. May C, Finch T. Implementing, embedding, and integrating practices: an outline of normalization process theory. *Sociology* 2009;43(3):535-54.
30. Greenhalgh T, Robert G, Macfarlane F, et al. Diffusion of innovations in service organizations: systematic review and recommendations. *The Milbank Quarterly* 2004;82(4):581-629.
31. Braun V, Clarke V. Using thematic analysis in psychology. *Qualitative research in psychology* 2006;3(2):77-101.
32. England N. Lessons learned review of the WannaCry Ransomware Cyber Attack. https://www.england.nhs.uk/wp-content/uploads/2018/02/lessons-learned-review-wannacry-ransomware-cyber-attack-cio-review.pdf, 2018.
33. News B. Facebook-Cambridge Analytica scandal https://www.bbc.co.uk/news/topics/c81zyn0888lt/facebook-cambridge-analytica-scandal2019 [cited 2019 10th December]. Available from: https://www.bbc.co.uk/news/topics/c81zyn0888lt/facebook-cambridge-analytica-scandal accessed 10th December 2019.
34. Whole Systems Partnership. Independent evaluation of Electronic Palliative Care Co-ordination Systems (EPaCCS) in England, 2016.

For peer review only

35. Hall S, Murchie P, Campbell C, et al. Introducing an electronic Palliative Care Summary (ePCS) in Scotland: patient, carer and professional perspectives. *Family practice* 2012;29(5):576-85.
36. Patterson R, Standing H, Lee M, et al. Paramedic information needs in end-of-life care: a qualitative interview study exploring access to a shared electronic record as a potential solution. *BMC Palliative Care* 2019;18(1):108. doi: 10.1186/s12904-019-0498-2
37. Dignam C, Thomas J, Brown M, et al. The impact of language on the interpretation of resuscitation clinical care plans by doctors. A mixed methods study. *PloS one* 2019;14(11)
38. Mack DS, Dosa D. Improving Advanced Care Planning through Physician Orders for Life-Sustaining Treatment (POLST) Expansion across the United States: Lessons Learned from State-Based Developments. *American Journal of Hospice and Palliative Medicine®* 2020;37(1):19-26. doi: 10.1177/1049909119851511
39. Zheng L, Finucane A, Oxenham D, et al. How good is primary care at identifying patients who need palliative care? A mixed-methods study. *European Journal of Palliative Care* 2013;20(5):216-22.

Funding statement

This paper summaries independent research funded by Connected Health Cities (CHC). The views expressed are those of the authors and not necessarily those of CHC.

Competing interests

We have no competing interests to declare.

Authors Contributions

HS, RP, ML, SD, MLh, AB, CE and KB (all authors) contributed to the design of the study. HS KB and RP conducted the data collection and analysis. HS and KB drafted the manuscript. All authors revised the article critically for important intellectual content. All authors approved the submitted draft.

Acknowledgements

We acknowledge the funder, the wider project team (led by Dr Kathryn Hall, clinical lead), Michele Spencer from North Tyneside Community and Health Care Forum for her input as patient and public representative and most importantly the participants who gave their time to the research.

Data Availability Statement

Given the sensitive nature of this qualitative research project the raw data generated and analysed during the current study are not publicly available to preserve the anonymity of participants. However, we are willing to make portions of transcripts (with any identifying information redacted) available upon reasonable request. Interested persons should contact the corresponding author.

For peer review only

May 2018

Interview Topic Guide- Health and social care professionals

Please note the interview topic guide is a living document. The design is iterative to tailor the interview to the needs of the participant, and the issues that they feel are most important. As analysis occurs concurrently the topic guide is likely to be adapted over time - questions might be added, amended or omitted – and used flexibly.

Section A: for interview

1. **Introduction**

- Thank you for agreeing to take part in this focus group. As you know, we are talking to health and social care professionals about their understandings, views and experiences of end of life care, and how it is managed.
- With your permission I would like to record the interview; all details will be confidential.
- Do you have any questions or concerns?
- *Obtain written consent.*

2. Management of end of life care

Could you talk me through how end of life care is typically managed?

Which health (and social) care professionals are involved?

Do all GPs have contact with palliative patients? How often? What is the role/responsibility of palliative leads?

(Gain insight into IT system: EMIS or SystmOne. Why this one?)

Our understanding is that 'palliative care' is when a terminal diagnosis has been given, and 'end of life care' refers to the last 3 days of life. Does this fit with your understanding or would you use other terminology?

What does end of life mean to you? Is there a difference between "approaching" end of life and 'the end'? How uncertain is this?

How is end of life care shared, communicated and co-ordinated between different health and social care professionals?

How does information flow between these groups?

At any point does this information flow feel particularly challenging?

What systems do you use in the delivery of end of life care?

How much can other professionals see/interact with the data you record?

How much can you see/interact with data recorded by others?

From your perspective, what works well/not so well in the pathway?

How does this vary depending on where the patient is? (Home/hospice/care home/hospital)

How successful do you feel the current system is in facilitating the patient's end of life wishes/preferences?

Example of a recent case where you feel it was handled well/not so well

How confident are you in the management of end of life care?

How confident are you in engaging patients, and their families, in discussions around end of life care?

For peer review only - http://bmjopen.bmj.com/site/about/guidelines.xhtml

Is there anything that would improve your confidence in discussing and managing end of life issues?

3. Sharing of information

Are you familiar with the deciding right documentation?
How frequently do you come into contact with these documents?

How familiar are you with emergency healthcare plans?
Who completes these?
What kind of information is recorded?

What patient health data would be useful for you when managing a patient at the end of life?
(e.g. recorded by others at other sites)

Are there any issues that you currently encounter related to patient health data (regarding access/consent etc.)?
What are the barriers/limitations in terms of systems/patient perceptions?

4. Electronic palliative care plan

Have you heard of EPaCCs? What is your understanding of this plan?

*****SHOW EPaCCS VIDEO*****

How do you think this care plan might integrate into current service provision?
Can you foresee any barriers? (e.g. usability/resources/time/confidentiality)
Explore access to/willingness to use mobile devices

What potential barriers/facilitators do you foresee related to the consenting and sharing of patient health data specifically?

5. Feedback

- *Thank you for your time.*
- *What made you take part in this interview?*
- *Are we asking the right questions?*
- *Do these questions allow you to talk about the most important issues for you?*
- *Is there anything else you think it would be useful for us to know?*

For peer review only - http://bmjopen.bmj.com/site/about/guidelines.xhtml

For peer review only

No	Item	Guide questions/description	Answer
**Domain 1: Research team and reflexivity**			
Personal Characteristics			
1.	Interviewer/facilitator	Which author/s conducted the interview or focus group?	Holly Standing, Rebecca Patterson, and Katie Brittain conducted the interviews<br>Page 6
2.	Credentials	What were the researcher's credentials? *E.g. PhD, MD*	Holly Standing, PhD, MSc, BSc<br>Rebecca Patterson – MSc, PGCert, BA (hons)<br>Katie Brittain - PhD, MA, BSc (hons)<br>Page 1
3.	Occupation	What was their occupation at the time of the study?	Holly Standing- Vice Chancellor's Research Fellow<br>Rebecca Patterson – Senior Research Assistant<br>Katie Brittain – Associate Professor of Ageing & Health
4.	Gender	Was the researcher male or female?	Female
5.	Experience and training	What experience or training did the researcher have?	All have significant experience of conducting interviews and focus groups

For peer review only

No	Item	Guide questions/description	Answer
Relationship with participants			
6.	Relationship established	Was a relationship established prior to study commencement?	Participants were known to the researcher via emails/phone calls prior to interview.<br>Page 6
7.	Participant knowledge of the interviewer	What did the participants know about the researcher? e.g. *personal goals, reasons for doing the research*	The participants had been provided with an information sheet about the research project and given the opportunity to ask the researchers any questions prior to interview.<br>Page 6
8.	Interviewer characteristics	What characteristics were reported about the interviewer/facilitator? e.g. *Bias, assumptions, reasons and interests in the research topic*	None
**Domain 2: study design**			
Theoretical framework			
9.	Methodological orientation and Theory	What methodological orientation was stated to underpin the study? *e.g. grounded theory, discourse analysis,*	Explorative qualitative study, thematic approach to analysis<br>Pages 5 and 6

For peer review only

No	Item	Guide questions/description	Answer
		ethnography, phenomenology, content analysis	
Participant selection			
10.	Sampling	How were participants selected? *e.g. purposive, convenience, consecutive, snowball*	Opportunistic sampling<br>Page 6
11.	Method of approach	How were participants approached? e.*g. face-to-face, telephone, mail, email*	Via email or mail<br>Page 6
12.	Sample size	How many participants were in the study?	71<br>Page 6 and 7
13.	Non-participation	How many people refused to participate or dropped out? Reasons?	We did not collect this information
Setting			
14.	Setting of data collection	Where was the data collected? e.*g. home, clinic, workplace*	University, public spaces (cafes), interviewees workplace, via the telephone<br>Page 6

For peer review only

No	Item	Guide questions/description	Answer
15.	Presence of non-participants	Was anyone else present besides the participants and researchers?	No
16.	Description of sample	What are the important characteristics of the sample? *e.g. demographic data, date*	62 health and social care professionals (GP, out-of-hours GPs, nurses, formal carers, paramedics, social workers, pharmacists, hospital doctors, other supporting professionals), 9 patients and family members (6 female, 3 male, 1 <60, 8 60+)<br>Page 6 and 7
Data collection			
17.	Interview guide	Were questions, prompts, guides provided by the authors? Was it pilot tested?	Interview schedules were used to structure the interviews, these were a living documents which were expanded throughout data collection to explore issues raised in earlier interviews.<br>Page 6 and supplementary material
18.	Repeat interviews	Were repeat interviews carried out? If yes, how many?	No
19.	Audio/visual recording	Did the research use audio or visual recording to collect the data?	Audio recording was used in all interviews<br>Page 6
20.	Field notes	Were field notes made during and/or after the interview or focus group?	Relevant notes were made during and after the interview<br>Page 8

For peer review only

No	Item	Guide questions/description	Answer
21.	Duration	What was the duration of the interviews or focus group?	Interviews between 22 and 93 mins.<br>Page 7
22.	Data saturation	Was data saturation discussed?	No
23.	Transcripts returned	Were transcripts returned to participants for comment and/or correction?	No
**Domain 3: analysis and findings**			
Data analysis			
24.	Number of data coders	How many data coders coded the data?	1 main coder (RP), 20% also coded by HS and KB<br>Page 8
25.	Description of the coding tree	Did authors provide a description of the coding tree?	No
26.	Derivation of themes	Were themes identified in advance or derived from the data?	Derived from the data
27.	Software	What software, if applicable, was used to manage the data?	Yes – Nvivo 12<br>Page 7

For peer review only

No	Item	Guide questions/description	Answer
28.	Participant checking	Did participants provide feedback on the findings?	No
Reporting			
29.	Quotations presented	Were participant quotations presented to illustrate the themes / findings? Was each quotation identified? e.*g. participant number*	Yes<br>Pages 8-16
30.	Data and findings consistent	Was there consistency between the data presented and the findings?	Yes<br>Pages 8-16
31.	Clarity of major themes	Were major themes clearly presented in the findings?	Yes<br>Pages 8-16
32.	Clarity of minor themes	Is there a description of diverse cases or discussion of minor themes?	No

For peer review only

BMJ Open

Information sharing challenges in end-of-life care: a qualitative study of patient, family and professional perspectives on the potential of an Electronic Palliative Care Coordination System

Journal:	*BMJ Open*
Manuscript ID	bmjopen-2020-037483.R2
Article Type:	Original research
Date Submitted by the Author:	28-Jul-2020
Complete List of Authors:	Standing, Holly ; Northumbria University, Department of Nursing Midwifery and Health<br>Patterson, Rebecca; Northumbria University, Research and Innovation Services<br>Lee, Mark; St Benedict's Hopsice and Specialist Palliative Care Centre<br>Dalkin, Sonia; Northumbria University Faculty of Health and Life Sciences, School of Health Education and Community Studies<br>Lhussier, Monique; Northumbria University Faculty of Health and Life Sciences, School of Health, Education and Community Studies<br>Bate, Angela; Northumbria University, Nursing, Midwifery and Health<br>Exley, Catherine; Newcastle University, Population Health Sciences Institute<br>Brittain, Katie; Newcastle University, Population Health Sciences Institute
**Primary Subject Heading**:	Qualitative research
Secondary Subject Heading:	Palliative care
Keywords:	Organisation of health services < HEALTH SERVICES ADMINISTRATION & MANAGEMENT, PALLIATIVE CARE, QUALITATIVE RESEARCH, Information technology < BIOTECHNOLOGY & BIOINFORMATICS

SCHOLARONE™
Manuscripts

I, the Submitting Author has the right to grant and does grant on behalf of all authors of the Work (as defined in the below author licence), an exclusive licence and/or a non-exclusive licence for contributions from authors who are: i) UK Crown employees; ii) where BMJ has agreed a CC-BY licence shall apply, and/or iii) in accordance with the terms applicable for US Federal Government officers or employees acting as part of their official duties; on a worldwide, perpetual, irrevocable, royalty-free basis to BMJ Publishing Group Ltd ("BMJ") its licensees and where the relevant Journal is co-owned by BMJ to the co-owners of the Journal, to publish the Work in this journal and any other BMJ products and to exploit all rights, as set out in our licence.

The Submitting Author accepts and understands that any supply made under these terms is made by BMJ to the Submitting Author unless you are acting as an employee on behalf of your employer or a postgraduate student of an affiliated institution which is paying any applicable article publishing charge ("APC") for Open Access articles. Where the Submitting Author wishes to make the Work available on an Open Access basis (and intends to pay the relevant APC), the terms of reuse of such Open Access shall be governed by a Creative Commons licence – details of these licences and which Creative Commons licence will apply to this Work are set out in our licence referred to above.

Other than as permitted in any relevant BMJ Author's Self Archiving Policies, I confirm this Work has not been accepted for publication elsewhere, is not being considered for publication elsewhere and does not duplicate material already published. I confirm all authors consent to publication of this Work and authorise the granting of this licence.

For peer review only

Information sharing challenges in end-of-life care: a qualitative study of patient, family and professional perspectives on the potential of an Electronic Palliative Care Coordination System

Holly C Standing[1], Rebecca Patterson[2], Mark Lee[3], Sonia Michelle Dalkin[4], Monique Lhussier[4], Angela Bate[1], Catherine Exley[5], and Katie Brittain[5]

[1]Department of Nursing, Midwifery and Health, Faculty of Health and Life Sciences, Northumbria University

[2]Research and Innovation Services, Northumbria University

[3]St Benedict's Hospice and Specialist Palliative Care Centre, Sunderland

[4]Department of Social Work, Education and Community Wellbeing, Faculty of Health and Life Sciences, Northumbria University

[5]Population Health Sciences Institute, Faculty of Medical Sciences, Newcastle University

Corresponding author: Dr Holly C Standing, Northumbria University, Coach Lane Campus West, Newcastle Upon Tyne, NE77AX (holly.standing@northumbria.ac.uk) 0191 232 6002

Word count 4,631

Abstract

Objectives To explore current challenges in inter-disciplinary management of end-of-life care in the community, and the potential of an Electronic Palliative Care Coordination System (EPaCCS) to facilitate the delivery of care which meets patient preferences.

Design Qualitative study using interviews and focus-groups.

Setting Health and Social Care Services in the North of England.

Participants 71 participants, 62 health and social care professionals, 9 patients and family members.

For peer review only

Results Four key themes were identified: information sharing challenges; information sharing systems; perceived benefits of an EPaCCS; barriers to use and requirements for an EPaCCS. Challenges in sharing information were a source of frustration for health and social care professionals as well as patients, and were suggested to result in inappropriate hospital admissions. Current systems were perceived by participants to not work well - paper advance care planning documentation was often unavailable or inaccessible, meaning it could not be used to inform decision-making at the point of care. Participants acknowledged the benefits of an EPaCCS to facilitate information sharing, however they also raised concerns about confidentiality, and availability of the increased time and resources required to access and maintain such a system.

Conclusions EPaCCS offer a potential solution to information sharing challenges in end-of-life care. However, our findings suggest that there are issues in the initiation and documentation of end-of-life discussions that must be addressed through investment in training in order to ensure that there is sufficient information regarding ACP to populate the system. There is a need for further qualitative research evaluating use of an EPaCCS, which explores benefits and challenges, uptake and reasons for disparities in use to better understand the potential utility and implications of such systems.

Keywords

Information technology, organisation of health services, palliative care, qualitative research

For peer review only

ARTICLE SUMMARY

Strengths and limitations of this study

- Large participant group from various stakeholder groups including health and social care practitioners, patients and carers
- This is the first study to include the experience of social workers and of a coroner
- Semi-structured interviews allowed us to collect rich descriptions of participants' experiences and perceptions of information sharing in end-of-life care and of an EPaCCS
- The study was conducted by a team of experienced researchers from a range of disciplinary backgrounds
- Data are from the pre-implementation phase of an EPaCCS trial, so findings are not drawn from direct experience of the system.

INTRODUCTION

The development of end-of-life care guidance in recent years supports attempts to allow dying persons more control over their end-of-life experience, through engagement of patients in Advance Care Planning (ACP).[1] ACP has been defined as 'a process that supports adults at any age or stage of health in understanding and sharing their personal values, life goals, and preferences regarding future medical care'. The goal of advance care planning is to help ensure that people receive medical care that is consistent with their values, goals and preferences during serious and chronic illness.'[2] ACP can include anyone involved in the provision of the patient's care, including nurses, doctors, care home managers and family members. Documents completed as part of ACP can include: a do not attempt cardiopulmonary resuscitation order (DNACPR), emergency health care plan (EHCP), and a written summary of the patients care preferences.[3] Paper versions of this documentation are completed and held by the patient to present to the health and social care professionals they

For peer review only

encounter.[4] ACP can facilitate the delivery of care which better reflects the patient's personal preferences,[1] and can have positive impacts on the person at the end-of-life and their family.[5] However, evidence suggests that the completion and use of ACP documentation is inconsistent.[6 7]

The delivery of high quality end-of-life care is an inter-professional endeavour, where the resources of various health and social care professionals must be combined for the benefit of the patient.[8] To achieve this, efficient inter-professional communication and information sharing across health and social care professionals involved in community end-of-life care is essential[9], but presents many challenges.[10-12] Accessibility of patient information can be limited, particularly for services who may only be episodically involved in end-of-life care but that offer crucial and timely support, such as paramedics, out-of-hours doctors, Admiral nurses (dementia specialist nurses), care home staff, and social workers.[13 14] Poor (or lack of) access to patient information can effect professionals' confidence in managing end-of-life patients, [13 14] family members satisfaction with care, [15] and patient outcomes.[5] Sharing of patient information is made more challenging by inconsistent documentation practices and systems which vary across organisations and professional boundaries.[16]

Electronic record systems offer a potential solution to these information sharing challenges.[17] A Framework for Action published by the National Information Board in 2014, details 2020 as a deadline for digital, real-time, and interoperable health records to be rolled out within the NHS.[18] Electronic Palliative care Co-ordinating Systems (EPaCCS) are an example of such an innovation. EPaCCS is an umbrella term covering a number of different electronic solutions that aim to capture patient wishes and preferred place of death and improve co-ordination of care in real time, through enabling the sharing of information across health and social care services. A 2013 Department of Health pilot evaluation in England estimated that EPaCCS could save £133,200 in hospital admissions per annum per 200,000 patient

For peer review only

population.[19] There are a number of versions of EPaCCS currently in use in the UK including the Key Information Summary (KIS) in Scotland,[20] Coordinate My Care in London,[21] South West EPaCCS[22] and Leeds EPaCCS[23]. Like many areas of the UK, the North East England does not yet have an EPaCCS in place. Research from other regions indicates that professional uptake and patient registration on such electronic systems is currently variable.[20 22] A systematic review found that 55% to 79% of patients with an EPaCCS died in their preferred place, higher than the general population.[24] The Scottish KIS has been suggested to be particularly beneficial for GPs working out of hours.[25] Challenges surrounding the use of an EPaCCS include professional reluctance to initiate end-of-life discussions,[6 26] increased burden from data entry for the primary users,[22] data ownership, funding, and consent.[27]

The aim of our study was to explore attitudes towards the potential of an EPaCCS solution for improving information sharing and coordination in end-of-life care within the North East of England. We provide an in-depth study of the professional and organisational factors that promote or inhibit the acceptability, usefulness and integration of collaborative care planning across health and social care into service delivery and everyday practice.[28-30]

METHODS

This study employed qualitative methods, involving semi-structured interviews and focus groups with patients, bereaved family members, and health and social care professionals.

Participants

A heterogeneous purposive sampling approach was employed to ensure representation from all health and social care professional groups who have a potential role in end-of-life care, and that a variety of perspectives and experiences of end-of-life care were sampled. Ethical approval for this study was obtained from the Health Research Authority (REC reference: 17/LO/2100).

For peer review only - http://bmjopen.bmj.com/site/about/guidelines.xhtml

Data collection

An interview schedule was developed by HS, RP and KB which included: current approaches to end-of-life care management, access to patient data, and attitudes towards introduction of an EPaCCS (including perceived benefits and challenges) an example interview schedule is provided in the supplementary material. A video demonstration of a draft version of the proposed EPaCCS was also used as a prompt during clinician interviews. The interview schedule evolved during the data collection process to allow iterative data analysis and further exploration of themes derived from the data. Informed consent was given by all participants prior to taking part. Interviews were digitally recorded.

Participants were recruited from an area within the North East of England where an EPaCCS was being trialled, data presented are from the pre-implementation phase of the trial. Patient and family participants were recruited through GP practices, practice staff acted as gatekeepers and sent information packs to patients on their palliative care registers and next of kin of deceased patients, those interested in participating returned a consent-to-contact form in a prepaid envelope. Clinicians were recruited through local clinical networks, calls for volunteers circulated via email, attendance of the study team at local commissioning meetings. Data collection took place from March to October 2018, interviews and focus groups were conducted at the participants' place of work or home, at university or community venues, or over the telephone, according to participant preference. Interviews and focus groups were conducted by HS, RP and KB. Clinician data were generated through 24 interviews and 5 focus groups. Two of the five focus groups were conducted with a mix of health and social care professionals including hospital doctors, pharmacists, out-of-hours GPs (n=9 and n=10). The remaining three focus groups were conducted with single profession groups: care home staff (n=5), social workers (n=5), nurse (n=9). Cancer was the primary diagnosis for all patient interviews and family interviews. Table 1 details participant

For peer review only

demographics. Clinician interviews ranged from 28-93 minutes, focus groups from 39-63 minutes, patients and family member interviews from 22 and 80 minutes. Field notes were collected before, during and after data collection. Data has been pseudonymised to protect participant identity.

Table 1: Participant demographics

**Health and social care professional role**	**No.**
GP	7
Out of hours GP	2
Nurse (including: Macmillan [cancer care nurse], Marie Curie [terminal care nurse], care home, district, rapid response, Admiral nurse [dementia care nurse])	21
Formal carer (care home)	3
Paramedic	10
Social worker	6
Pharmacist	4
Hospital doctor	4
Other supporting professions (including: care coordinator, Macmillan manager, physiotherapist, coroner, palliative care team)	5
**Patient/family**	
Patient	7 (4 female, 5 lived alone, 6 60+ years old)
Family	2 (both female, 60+)
**Total**	**71**

Data Analysis

Audio recordings were transcribed verbatim by an independent transcription company; transcripts were checked for accuracy by (HS, RP, KB). The data management software NVivo 12 was used to develop and refine a coding scheme.

For peer review only

We adopted an iterative approach to data analysis. Data collection and analysis ran concurrently throughout the study and analysis of early transcripts informed the refinement of the interview schedule for later interviews. The five-step process of thematic analysis [31] was adopted to develop and refine codes and themes from the data. Field notes taken during data collection were utilised in data analysis to enhance the reflexive process. Reflexivity is the process of accounting for the situatedness of the researcher within the research and the potential effect of this on the data collected and interpretation.[32] Credibility measures included a process of continuous review and discussion of themes at group analysis sessions, and at wider team meetings. Three researchers (HS, RP, KB) independently coded 20% of the transcripts before coming together to compare codes and discuss discrepancies.

Patient and public involvement

The study was supported by a patient and public involvement representative who contributed to the design and development of the study, advised on recruitment processes, and participant information and consent materials.

FINDINGS

Four themes were identified through data analysis: information sharing challenges; information sharing systems; perceived benefits of an EPaCCS; barriers to use and requirements for an EPaCCS. These are presented in turn below, with illustrative quotations.

Information sharing challenges

Participants were keen for end-of-life patients in the community to be managed at home and avoid inappropriate hospital admissions where possible, however, poor communication and information sharing between services was a barrier to achieving this goal. Paramedics and out-of-hours GPs may be alerted by call handlers that the patient they are attending is at the end-of-life, but often were going in ‘blind’ as they had no prior access to the patient’s

For peer review only

condition or care preferences. Without access to up-to-date patient information, paramedics felt that in such situations they had no choice but to admit the patient:

> *The number of times I've said, "They are going to have to go in. I don't want to take them in, but I don't have a choice here, because I don't have the information." If you don't have the information, you can't make an informed decision, so to leave them at home would be the wrong thing to do, even though you know morally you'd like to.* (Paramedic6)

Inappropriate hospital admission of end-of-life patients was also a source of frustration for GPs who suggested that these could be avoided if they were contacted for advice:

> *I suppose the admissions where they're palliative are the ones that I get annoyed about […] they get admitted, and if somebody had asked you, you know, "What should we do with this lady?"* [we could advise on management but]*that's not always easy because we don't work every day. We are obviously not working in the evenings or the weekends*[...] (GP6)

Indeed, the continuity of care afforded by the GP role means they often have established relationships with end-of-life patients and would most likely have access to detailed information regarding their condition, history and care preferences, which should inform decision making in any circumstance. However as in-hours GPs are not available 24/7 there is a need for patient information to be stored in a manner that is easily accessible to all services who may require it. Paramedics or out-of hours GPs are unlikely to have had prior contact when called out to an end-of-life patient, and would benefit to access to greater

For peer review only

patient information to facilitate their management of such patients, and potentially avoid hospital admissions.

Poor communication and information sharing could have negative impacts on the patient and their family. Where patient information is unavailable, and services were going in ‘blind’ the burden of information sharing falls on the patient or their family.

> *Whatever doctor I would see, they wouldn't know a lot about me, so I'd have to constantly start from the beginning* […family] *couldn't come into the meetings with me because they'd get so upset because they'd watch me explain everything from the start. I think that was worse than hearing any bad news* (Patient5)

Having to repeatedly reiterate their story could be distressing for the patient and their family, and could also damage the professional patient relationship; as the patient above describes this constant repeating of her story led her family to disengage from encounters with health and social care professionals.

Lack of information sharing mean it is not always clear who should be responsible for verifying death and issuing the death certificate. This could result in the death being designated as ‘unexpected’ and the coroners being inappropriately involved, causing unnecessary delays and distress to the family:

> *when they've gone home expecting that* [the patient will die] *and then all of a sudden somebody says, "We don't know who can issue the certificate because the GP hasn't seen them because it was the out of hours doctor* [who came]…" *and it takes hours to sort it out*. (Coroner1)

Information sharing systems

For peer review only

Information about patients at the end-of-life is primarily stored in two formats: 1) paper-based ACP documents which are held by the patient, and 2) isolated patient record systems (as it currently stands) versus an integrated system that can be updated and available to all professionals (and patients in 'real time').

Paper ACP documents facilitate information sharing, however, their usefulness is largely dependent on the patient, or their family, having the documents ready to hand or easily accessible and up to date. Many participants indicated this was often not the case:

> *you're meant to have a bright yellow envelope on your mantle or on your sideboard. So, if you go in as a doctor, or if you go in as an ambulance crew, you know it's there, and it's bright, illuminous yellow, but the number of folks, "Oh, I've got one of those," and it's under four piles of stuff, or it's in their bedside cabinet. It's like, "Well, no-one's going to respect that," because no-one's going to spend 10 minutes looking for it if you've arrested. They're going to start resus.* (Out-of-hours GP1)

If the documentation is not available, either misplaced or forgotten, then health and social care professionals have to proceed as if it did not exist.

Having a standardised location for the documentation in the patient's home (such as the mantelpiece, for example) may improve access to this information. However, keeping the documentation in a visible location may also be a potential source of distress for families. Whilst some patient participants were comfortable having theirs visible, others chose to keep them hidden as they acted as a constant reminder of the imminent death.

> *I had put the yellow envelope in my knicker drawer* […] *They said, "Put it up there."* [mantel piece] *So, I said, "No, I'm not putting it up there. I don't want to look at that all the time."* (Bereaved family2)

For peer review only

In addition to paper versions of ACP documentation where available, health and social care services utilise electronic patient record systems to record and manage the information that they collect. These systems tend to work in isolation, and are generally not inter-operable, meaning there is no simple way to share information with others. Many patients assumed that their information was being shared, and they did not necessarily perceive services as discrete organisations, rather part of a united healthcare system. Although patients recognised that protecting patient confidentially was important, the benefits of sharing information were perceived to significantly outweigh these concerns.

> *What if you travel? What if you're visiting round the country? You don't know what's going to happen* […] *I went to London a couple of times when I was ill. What happens if something happens to me down there? How long would it take them to find anything out about me? In the meantime, what's happening to me?* (Patient5)

Perceived benefits of an EPaCCS

Participants identified several potential benefits of adopting an EPaCCS. NHS electronic patient records systems were perceived by the majority of participants, both patient and professional, as being extremely disjointed and often outdated.

> *I certainly think there should be a national set-up. Everybody should be reading from the same sheet, yes.* (Patient6)

Patients welcomed a system that would facilitate better transfer of information between services so that they would no longer have to act as the conduit for passing information between services by repeating their story.

For peer review only

Professionals indicated that an EPaCCS may save time and facilitate the provision of care that was better aligned with patients' wishes. An EPaCCS could potentially allow multiple users to simultaneously access and update a patient's record, ensuring changes to the patient's condition and/or care plan are immediately accessible to all professionals involved in their care.

> *I think the thing is, when it's a paper copy there's only one actual current live copy that is up to date* […] *the electronic copy means multiple users could be using it at the same time.* (Nurse6)

An EPaCCS was suggested to be particularly beneficial to services without sustained relationships with end-of-life patients who were often 'information poor'. Improved access to patient information could potentially increase the confidence of these services in delivering end-of-life care.

> *You do get very limited information and you're going into a person to talk to them about what care they need with very limited information. And sometimes when they are end-of-life, the last thing they want to talk about is how they're going to die and why they're going to die* (Social Worker5)

Barriers to use, and requirements for an EPaCCS

Professional participants did not express universal enthusiasm for the introduction of an EPaCCS. Paramedics and out-of-hours GPs were generally positive about the potential of the system, as discussed above, however GPs were less enthusiastic; the system was not necessarily perceived to offer improvements over current approaches to information storing and recording. GPs expressed concern about implications of the system for their workload;

For peer review only

GPs are often the most 'information rich' meaning responsibility for inputting data into the system would fall on them. If the proposed EPaCCS was not fully inter-operable with existing systems it would result in duplication of tasks and increased demands on GP time.

> If it's [a] *separate* [system] *then you'd be duplicating because you'd need to add it to the system wouldn't you?* […] *You'd find GPs wouldn't engage with it, because it would just be an extra thing they have to do.* (GP3)

The implications of a shared EPaCCS on maintaining data protection and the security of patient information was a major concern for many of the professional participants.

> *We're still recovering from a cyber-attack a year ago, and there are still some documents that we can't open* […] *so it has to be encrypted to a level that it's safe* (Hospital doctor3)

Such concerns are understandable given recent security threats [33] and scandals involving misuse of personal data.[34] However, this did not appear to be a concern for patients, many of whom assumed that data sharing practices already occurred and viewed moves to facilitate information sharing through electronic records with the aim of improving care as welcome and necessary progress.

Incorporating the informational requirements of all health and social care groups into one system was highlighted as a challenge; different roles have different informational needs. For example, nurses indicated that lists of patient medication are essential information for their practice to ensure they are not prescribing contraindicated medications. Catering for individualised requirements such as these risks the EPaCCS becoming overwhelming and challenging to navigate.

For peer review only

It might just slow people down. It will take longer actually to get through cases because you have to read through them [patient notes] (Out-of-hours GP1)

Having to sift through irrelevant information to find what is germane to their role may become burdensome. An EPaCCS with extensive information about the patient and limited in its ability to filter efficiently may not be preferable to paper ACP documentation being available upon arrival.

Professionals who spend most of their time working remotely, doing home visits, may gain the most benefit from an EPaCCS.

electronic information, that would solve the main problems that we have, which is lack of information at times. So yes, that would be great. (Paramedic5)

However, an EPaCCS needs to be accessible to these professionals. If an EPaCCS cannot be accessed and updated by those working in the community, the information contained within will quickly cease to be up-to-date. Successful uptake of an EPaCCS is also dependent upon the technology available to access the system. Electronic devices available to those working in the community were reported to often be of low specification, raising doubts about likelihood that they would be able to support accessibility.

It also has to be an 'on the go' portal, you were saying how busy you are and so it has to work on a tablet or on an iPad because a lot of this work is being inputted in the community. You can't rely on someone going back to their office, after they've done five house calls or five social work visits or

For peer review only

five whatever [..] *It has to be a real-time and easy to access.* (Hospital Doctor2)

A need for ongoing support for the EPaCCS following the implementation period was highlighted. Participants expressed concern that investing time learning a new system would be wasted if ongoing infrastructure was not available to support its continued use.

I've done many projects before within health, and there's a massive emphasis placed at the beginning of every project and they put it on. Then, afterwards, staff move away, because it's not part of their project and we're suddenly left with it. The system is changing and evolving, but there's not always the infrastructure in place to support us to update it. I think that's a real concern. (Nurse19)

Implementation of an EPaCCS was not necessarily perceived to solve the challenges in inter-disciplinary management of end-of-life care alone. Access to ACP documentation will only be useful if the documentation itself is completed to a satisfactory standard, if insufficient detail is provided it cannot be used to inform decision making. Paramedics and out-of-hours doctors indicated that ACP documentation that they encountered was of a variable standard, and these documents should be completed with these services in mind as the intended reader.

I'm making a decision within 30 seconds I've walked through the door, "Am I going to attempt to save a life or am I going to let them expire?" The only thing between me and setting off a treatment path is the end-of-life care plan, so it needs to make perfect sense.(Paramedic4)

For peer review only

DISCUSSION

Principle statement of findings

This study provides insight into current challenges in information sharing in end-of-life care, which were recognised as a barrier to effective end-of-life care, and the potential of an EPaCCS system to overcome these challenges. Inconsistent information sharing within and between health and social care services was a source of frustration for patients, paper based ACP documentation does not appear to be an effective system for recording and sharing patients' care preferences; often these documents are unavailable when required and are variable in quality. Patients welcomed the idea of an EPaCCS that facilitated sharing of their information between health and social care services, they did not report the same concerns about data protection and security issues that concerned clinicians. Both groups of participants perceived the potential for improvements in delivery aligned with patient's wishes from adoption of an EPaCCS. Perceived barriers to the successful implementation of an EPaCCS were focused on increased demand on time and the lack of infrastructure in place to support the system. Care must be taken to ensure that information contained within the EPaCCS does not become overwhelming. This is particularly important for emergency services, such as paramedics, who work under extreme time pressures limiting their ability to access and digest vast amounts of information. Introduction of an EPaCCS was not seen to resolve current challenges in management of patients at the end-of-life without investment into improving the frequency with which ACP documentation is completed and the quality of the information recorded. Paramedics in particular suggested their informational needs must be a priority to ensure ACP documentation is completed to a standard where it can be used to inform decision making.

For peer review only

Strengths and weaknesses

A key strength of this study is the large sample which includes a wide range of health and social care professionals involved in end-of-life care, the majority of previous qualitative studies have employed small samples,[35 36] and ours is the first study to our knowledge to include the perspectives of supporting professions such as social workers and coroners. In addition, our study included a greater number of participants from out-of-hours services than previous qualitative studies.[22 36] As the data were collected during the developmental pre-implementation phase of an EPaCCS trial, participants were only able to reflect hypothetically on the impacts of such a system on their practice rather than drawing on direct experiences of working with the system. In line with previous EPaCCS studies, a second limitation of the study stems from the recruitment process for patients and family members, which was, operationalised through GP practices. Staff thus acted as gatekeepers and may have been selective in who they referred for participation.

Comparison with other work

There are only a limited number of qualitative studies exploring attitudes towards and use of EPaCCS.[22 35-37] Our findings support those of a recent systematic review which identified the burden of inputting data and IT systems as the main challenge to implementation of EPaCCS.[24] GP participants expressed a reluctance to dedicate time to inputting data into the EPaCCS, this has been observed in previous studies where GPs have reported delegating much of this work to administrative staff.[22] However, there is high use of the Scottish KIS system despite reliance on GPs to populate.[20]

A key finding of our study was that introduction of an EPaCCS alone does not provide a solution to some of the current difficulties regarding inter-disciplinary management of end-of-life patients in the community. An EPaCCS will only facilitate access to patient

For peer review only

information, the utility of which is largely dependent upon its quality; improving access to ACP documentation is not going to improve care if the information recorded within them is of poor quality or insufficient detail, or has not been completed. This demonstrates a need for investment in training for health and social care professionals in ACP discussions and documentation to instil confidence and improve quality. Inconsistencies in ACP documentation have also been observed within an Australian healthcare context.[38]

This article highlights concerns regarding the infrastructure required to support an EPaCCS including: demand on already stretched professionals, technology provision required to enable access, and continued support to ensure the system is updated. Ensuring appropriate infrastructure is in place has emerged as a challenge in the implementation of coordinated records for end-of-life patients in both UK and US contexts.[24 39] As this article focuses on the pre-implementation stage of an EPaCCS trial future research should explore the long-term uptake and impact of an EPaCCS to better understand the infrastructure required to stain such a system. Although there have been recent mixed methods studies conducted post-implementation of an EPaCCS,[20 25 40] there is a need for further qualitative research exploring the benefits and challenges experienced whilst using the system for all stakeholders including in-hours and out-of-hours services, and in particular on the impacts of the experiences of patients and families. High and low users of the system should be explored to identify reasons for disparities and potential ways of promoting use of the system.

Conclusions

EPaCCS may offer a potential solution to information sharing difficulties in the inter-disciplinary management of end-of-life care. However, implementation of an EPaCCS alone will not improve management of patients at the end-of-life it will only facilitate the access to patient information. Introduction of EPaCCS must be accompanied by investment in training

For peer review only

around communication skills focusing on the initiation of end-of-life discussions and effective completion of ACP documentation.

REFERENCES

1. NICE. Advance Care Planning: Emergency and acute medical care in over 16s: service delivery and organisation. 2018
2. Sudore RL, Lum HD, You JJ, et al. Defining Advance Care Planning for Adults: A Consensus Definition From a Multidisciplinary Delphi Panel. *J Pain Symptom Manage* 2017;53(5):821-32.e1. doi: 10.1016/j.jpainsymman.2016.12.331 [published Online First: 2017/01/08]
3. Northern Cancer Alliance. Deciding right Regional forms https://www.northerncanceralliance.nhs.uk/deciding-right/deciding-right-regional-forms/2019 [cited 2019 6th November]. Available from: https://www.northerncanceralliance.nhs.uk/deciding-right/deciding-right-regional-forms/ accessed 6th November 2019.
4. Mullick A, Martin J, Sallnow L. An introduction to advance care planning in practice. *Bmj* 2013;347:f6064.
5. Detering KM, Hancock AD, Reade MC, et al. The impact of advance care planning on end of life care in elderly patients: randomised controlled trial. *BMJ* 2010;340 doi: 10.1136/bmj.c1345
6. Dalkin SM, Lhussier M, Philipson P, et al. Reducing inequalities in care for patients with non-malignant diseases: Insights from a realist evaluation of an integrated palliative care pathway. *Palliative Medicine* 2016;30(7):690-97. doi: 10.1177/0269216315626352
7. National Institute for Health and Care Excellence. Chapter 15 Advance Care Planning. Emergency and acute medical care in over 16s: service delivery and organisation. Nice guideline 94 ed, 2018:1-39.
8. Huby G, Harris FM, Powell AE, et al. Beyond professional boundaries: relationships and resources in health services' modernisation in England and Wales. *Sociology of Health & Illness* 2014;36(3):400-15. doi: doi:10.1111/1467-9566.12067
9. Tanabe K, Sawada K, Shimada M, et al. Evaluation of A Novel Information-Sharing Instrument for Home-Based Palliative Care. *American Journal of Hospice & Palliative Medicine* 2015;32(6):611-19. doi: 10.1177/1049909114533141
10. Powell AE, Davies HT. The struggle to improve patient care in the face of professional boundaries. *Social science & medicine* 2012;75(5):807-14.
11. Kreindler SA, Dowd DA, Dana Star N, et al. Silos and Social Identity: The Social Identity Approach as a Framework for Understanding and Overcoming Divisions in Health Care. *The Milbank Quarterly* 2012;90(2):347-74. doi: doi:10.1111/j.1468-0009.2012.00666.x
12. Braithwaite J, Clay-Williams R, Vecellio E, et al. The basis of clinical tribalism, hierarchy and stereotyping: a laboratory-controlled teamwork experiment. *BMJ Open* 2016;6(7) doi: 10.1136/bmjopen-2016-012467
13. Kirk A, Crompton PW, Knighting K, et al. Paramedics and their role in end-of-life care: perceptions and confidence. *Journal of Paramedic Practice* 2017;9(2):71-79.
14. Murphy-Jones G, Timmons S. Paramedics' experiences of end-of-life care decision making with regard to nursing home residents: an exploration of influential issues and factors. *Emergency Medicine Journal* 2016;33(10):722-6.
15. Thompson GN, Menec VH, Chochinov HM, et al. Family satisfaction with care of a dying loved one in nursing homes: what makes the difference? *Journal of Gerontological Nursing* 2008;34(12):37-44. doi: 10.3928/00989134-20081201-10

16. Street A, Blackford J. Communication issues for the interdisciplinary community palliative care team. *J CLIN NURS* 2001;10(5):643.
17. NHS England. Actions for End of Life Care: 2014-16. In: England N, ed. Leeds: NHS England, 2014.
18. NHS National Information Board. Personalised Health and care 2020: Using Data and Technology to Transform Outcomes for Patients and Citizens. In: Board NNI, ed., 2014.
19. NHS Improving Quality. Economic Evaluation of the Electronic Palliative Care Coordination System (EPaCCS) Early Implementer Sites, 2013.
20. Finucane AM, Davydaitis D, Horseman Z, et al. Electronic care coordination systems for people with advanced progressive illness: a mixed-methods evaluation in Scottish primary care. *British Journal of General Practice* 2019:bjgp19X707117. doi: 10.3399/bjgp19X707117
21. Smith C, Hough L, Cheung C-C, et al. Coordinate My Care: a clinical service that coordinates care, giving patients choice and improving quality of life. *BMJ supportive & palliative care* 2012;2(4):301-07.
22. Wye L, Lasseter G, Simmonds B, et al. Electronic palliative care coordinating systems (EPaCCS) may not facilitate home deaths: A mixed methods evaluation of end of life care in two English counties. *Journal of Research in Nursing* 2016;21(2):96-107. doi: 10.1177/1744987116628922
23. Henry K, Kite S, McDermott S, et al. THE DEVELOPMENT OF AN ELECTRONIC REPORTING MECHANISM TO SUPPORT THE USE OF EPACCS IN CLINICAL PRACTICE. *BMJ Supportive & Palliative Care* 2014;4(Suppl 1):A65-A66. doi: 10.1136/bmjspcare-2014-000654.186
24. Leniz J, Weil A, Higginson IJ, et al. Electronic palliative care coordination systems (EPaCCS): a systematic review. *BMJ Supportive and Palliative Care* 2019:bmjspcare-2018-001689. doi: 10.1136/bmjspcare-2018-001689
25. Tapsfield J, Hall C, Lunan C, et al. Many people in Scotland now benefit from anticipatory care before they die: an after death analysis and interviews with general practitioners. *BMJ Support Palliat Care* 2019;9(4):e28. doi: 10.1136/bmjspcare-2015-001014 [published Online First: 2016/04/15]
26. Dalkin S, Lhussier M, Jones D, et al. Open communication strategies between a triad of 'experts' facilitates death in usual place of residence: A realist evaluation. *Palliative Medicine* 2018;32(5):980-89. doi: 10.1177/0269216318757132
27. NHS Improving Quality. Electronic Palliative Care Co-ordination Systems (EPaCCS) in England: Survey of clinical commissioning groups (2013) by the National End of Life Care Intelligence Network. In: England PH, ed. Public Health England, 2013.
28. Oakley A, Strange V, Bonell C, et al. Process evaluation in randomised controlled trials of complex interventions. *Bmj* 2006;332(7538):413-16.
29. May C, Finch T. Implementing, embedding, and integrating practices: an outline of normalization process theory. *Sociology* 2009;43(3):535-54.
30. Greenhalgh T, Robert G, Macfarlane F, et al. Diffusion of innovations in service organizations: systematic review and recommendations. *The Milbank Quarterly* 2004;82(4):581-629.
31. Braun V, Clarke V. Using thematic analysis in psychology. *Qualitative research in psychology* 2006;3(2):77-101.
32. Berger R. Now I see it, now I don't: researcher's position and reflexivity in qualitative research. *Qualitative Research* 2015;15(2):219-34. doi: 10.1177/1468794112468475
33. England N. Lessons learned review of the WannaCry Ransomware Cyber Attack. https://www.england.nhs.uk/wp-content/uploads/2018/02/lessons-learned-review-wannacry-ransomware-cyber-attack-cio-review.pdf, 2018.
34. News B. Facebook-Cambridge Analytica scandal https://www.bbc.co.uk/news/topics/c81zyn0888lt/facebook-cambridge-analytica-scandal2019 [cited 2019 10th December]. Available from: https://www.bbc.co.uk/news/topics/c81zyn0888lt/facebook-cambridge-analytica-scandal accessed 10th December 2019.

For peer review only

35. Whole Systems Partnership. Independent evaluation of Electronic Palliative Care Co-ordination Systems (EPaCCS) in England, 2016.
36. Hall S, Murchie P, Campbell C, et al. Introducing an electronic Palliative Care Summary (ePCS) in Scotland: patient, carer and professional perspectives. *Family practice* 2012;29(5):576-85.
37. Patterson R, Standing H, Lee M, et al. Paramedic information needs in end-of-life care: a qualitative interview study exploring access to a shared electronic record as a potential solution. *BMC Palliative Care* 2019;18(1):108. doi: 10.1186/s12904-019-0498-2
38. Dignam C, Thomas J, Brown M, et al. The impact of language on the interpretation of resuscitation clinical care plans by doctors. A mixed methods study. *PloS one* 2019;14(11)
39. Mack DS, Dosa D. Improving Advanced Care Planning through Physician Orders for Life-Sustaining Treatment (POLST) Expansion across the United States: Lessons Learned from State-Based Developments. *American Journal of Hospice and Palliative Medicine®* 2020;37(1):19-26. doi: 10.1177/1049909119851511
40. Zheng L, Finucane A, Oxenham D, et al. How good is primary care at identifying patients who need palliative care? A mixed-methods study. *European Journal of Palliative Care* 2013;20(5):216-22.

Funding statement

This paper summaries independent research funded by Connected Health Cities (CHC). The views expressed are those of the authors and not necessarily those of CHC.

Competing interests

We have no competing interests to declare.

Authors Contributions

HS, RP, ML, SD, MLh, AB, CE and KB (all authors) contributed to the design of the study. HS KB and RP conducted the data collection and analysis. HS and KB drafted the manuscript. All authors revised the article critically for important intellectual content. All authors approved the submitted draft.

Acknowledgements

We acknowledge the funder, the wider project team (led by Dr Kathryn Hall, clinical lead), Michele Spencer from North Tyneside Community and Health Care Forum for her input as patient and public representative and most importantly the participants who gave their time to the research.

Data Availability Statement

Given the sensitive nature of this qualitative research project the raw data generated and analysed during the current study are not publicly available to preserve the anonymity of participants. However, we are willing to make portions of transcripts (with any identifying information redacted) available upon reasonable request. Interested persons should contact the corresponding author.

For peer review only

May 2018

Interview Topic Guide- Health and social care professionals

Please note the interview topic guide is a living document. The design is iterative to tailor the interview to the needs of the participant, and the issues that they feel are most important. As analysis occurs concurrently the topic guide is likely to be adapted over time - questions might be added, amended or omitted – and used flexibly.

Section A: for interview

1. **Introduction**

- Thank you for agreeing to take part in this focus group. As you know, we are talking to health and social care professionals about their understandings, views and experiences of end of life care, and how it is managed.
- With your permission I would like to record the interview; all details will be confidential.
- Do you have any questions or concerns?
- *Obtain written consent.*

2. Management of end of life care

Could you talk me through how end of life care is typically managed?

Which health (and social) care professionals are involved?

Do all GPs have contact with palliative patients? How often? What is the role/responsibility of palliative leads?

(Gain insight into IT system: EMIS or SystmOne. Why this one?)

Our understanding is that 'palliative care' is when a terminal diagnosis has been given, and 'end of life care' refers to the last 3 days of life. Does this fit with your understanding or would you use other terminology?

What does end of life mean to you? Is there a difference between "approaching" end of life and 'the end'? How uncertain is this?

How is end of life care shared, communicated and co-ordinated between different health and social care professionals?

How does information flow between these groups?

At any point does this information flow feel particularly challenging?

What systems do you use in the delivery of end of life care?

How much can other professionals see/interact with the data you record?

How much can you see/interact with data recorded by others?

From your perspective, what works well/not so well in the pathway?

How does this vary depending on where the patient is? (Home/hospice/care home/hospital)

How successful do you feel the current system is in facilitating the patient's end of life wishes/preferences?

Example of a recent case where you feel it was handled well/not so well

How confident are you in the management of end of life care?

How confident are you in engaging patients, and their families, in discussions around end of life care?

For peer review only - http://bmjopen.bmj.com/site/about/guidelines.xhtml

Is there anything that would improve your confidence in discussing and managing end of life issues?

3. Sharing of information

Are you familiar with the deciding right documentation?
How frequently do you come into contact with these documents?

How familiar are you with emergency healthcare plans?
Who completes these?
What kind of information is recorded?

What patient health data would be useful for you when managing a patient at the end of life?
(e.g. recorded by others at other sites)

Are there any issues that you currently encounter related to patient health data (regarding access/consent etc.)?
What are the barriers/limitations in terms of systems/patient perceptions?

4. Electronic palliative care plan

Have you heard of EPaCCs? What is your understanding of this plan?

*****SHOW EPaCCS VIDEO*****

How do you think this care plan might integrate into current service provision?
Can you foresee any barriers? (e.g. usability/resources/time/confidentiality)
Explore access to/willingness to use mobile devices

What potential barriers/facilitators do you foresee related to the consenting and sharing of patient health data specifically?

5. Feedback

- *Thank you for your time.*
- *What made you take part in this interview?*
- *Are we asking the right questions?*
- *Do these questions allow you to talk about the most important issues for you?*
- *Is there anything else you think it would be useful for us to know?*

For peer review only - http://bmjopen.bmj.com/site/about/guidelines.xhtml

No	Item	Guide questions/description	Answer
**Domain 1: Research team and reflexivity**			
Personal Characteristics			
1.	Interviewer/facilitator	Which author/s conducted the interview or focus group?	Holly Standing, Rebecca Patterson, and Katie Brittain conducted the interviews<br>Page 6
2.	Credentials	What were the researcher's credentials? *E.g. PhD, MD*	Holly Standing, PhD, MSc, BSc<br>Rebecca Patterson – MSc, PGCert, BA (hons)<br>Katie Brittain - PhD, MA, BSc (hons)<br>Page 1
3.	Occupation	What was their occupation at the time of the study?	Holly Standing- Vice Chancellor's Research Fellow<br>Rebecca Patterson – Senior Research Assistant<br>Katie Brittain – Associate Professor of Ageing & Health
4.	Gender	Was the researcher male or female?	Female
5.	Experience and training	What experience or training did the researcher have?	All have significant experience of conducting interviews and focus groups

For peer review only

No	Item	Guide questions/description	Answer
Relationship with participants			
6.	Relationship established	Was a relationship established prior to study commencement?	Participants were known to the researcher via emails/phone calls prior to interview.<br>Page 6
7.	Participant knowledge of the interviewer	What did the participants know about the researcher? e.g. *personal goals, reasons for doing the research*	The participants had been provided with an information sheet about the research project and given the opportunity to ask the researchers any questions prior to interview.<br>Page 6
8.	Interviewer characteristics	What characteristics were reported about the interviewer/facilitator? e.g. *Bias, assumptions, reasons and interests in the research topic*	None
**Domain 2: study design**			
Theoretical framework			
9.	Methodological orientation and Theory	What methodological orientation was stated to underpin the study? *e.g. grounded theory, discourse analysis,*	Explorative qualitative study, thematic approach to analysis<br>Pages 5 and 6

For peer review only

No	Item	Guide questions/description	Answer
		ethnography, phenomenology, content analysis	
Participant selection			
10.	Sampling	How were participants selected? *e.g. purposive, convenience, consecutive, snowball*	Opportunistic sampling<br>Page 6
11.	Method of approach	How were participants approached? e.*g. face-to-face, telephone, mail, email*	Via email or mail<br>Page 6
12.	Sample size	How many participants were in the study?	71<br>Page 6 and 7
13.	Non-participation	How many people refused to participate or dropped out? Reasons?	We did not collect this information
Setting			
14.	Setting of data collection	Where was the data collected? e.*g. home, clinic, workplace*	University, public spaces (cafes), interviewees workplace, via the telephone<br>Page 6

For peer review only

No	Item	Guide questions/description	Answer
15.	Presence of non-participants	Was anyone else present besides the participants and researchers?	No
16.	Description of sample	What are the important characteristics of the sample? *e.g. demographic data, date*	62 health and social care professionals (GP, out-of-hours GPs, nurses, formal carers, paramedics, social workers, pharmacists, hospital doctors, other supporting professionals), 9 patients and family members (6 female, 3 male, 1 <60, 8 60+)<br>Page 6 and 7
Data collection			
17.	Interview guide	Were questions, prompts, guides provided by the authors? Was it pilot tested?	Interview schedules were used to structure the interviews, these were a living documents which were expanded throughout data collection to explore issues raised in earlier interviews.<br>Page 6 and supplementary material
18.	Repeat interviews	Were repeat interviews carried out? If yes, how many?	No
19.	Audio/visual recording	Did the research use audio or visual recording to collect the data?	Audio recording was used in all interviews<br>Page 6
20.	Field notes	Were field notes made during and/or after the interview or focus group?	Relevant notes were made during and after the interview<br>Page 8

For peer review only

No	Item	Guide questions/description	Answer
21.	Duration	What was the duration of the interviews or focus group?	Interviews between 22 and 93 mins.<br>Page 7
22.	Data saturation	Was data saturation discussed?	No
23.	Transcripts returned	Were transcripts returned to participants for comment and/or correction?	No
**Domain 3: analysis and findings**			
Data analysis			
24.	Number of data coders	How many data coders coded the data?	1 main coder (RP), 20% also coded by HS and KB<br>Page 8
25.	Description of the coding tree	Did authors provide a description of the coding tree?	No
26.	Derivation of themes	Were themes identified in advance or derived from the data?	Derived from the data
27.	Software	What software, if applicable, was used to manage the data?	Yes – Nvivo 12<br>Page 7

For peer review only

No	Item	Guide questions/description	Answer
28.	Participant checking	Did participants provide feedback on the findings?	No
Reporting			
29.	Quotations presented	Were participant quotations presented to illustrate the themes / findings? Was each quotation identified? e*.g. participant number*	Yes<br>Pages 8-16
30.	Data and findings consistent	Was there consistency between the data presented and the findings?	Yes<br>Pages 8-16
31.	Clarity of major themes	Were major themes clearly presented in the findings?	Yes<br>Pages 8-16
32.	Clarity of minor themes	Is there a description of diverse cases or discussion of minor themes?	No

For peer review only
